# Reduced Scaling of Optimal Regional Orbital Localization via Sequential Exhaustion of the Single-Particle Space

**DOI:** 10.1021/acs.jctc.2c00315

**Published:** 2022-07-11

**Authors:** Guorong Weng, Mariya Romanova, Arsineh Apelian, Hanbin Song, Vojtěch Vlček

**Affiliations:** † Department of Chemistry and Biochemistry, University of California, Santa Barbara, California 93106-9510, United States; ‡ Department of Materials, University of California, Santa Barbara, California 93106-9510, United States

## Abstract

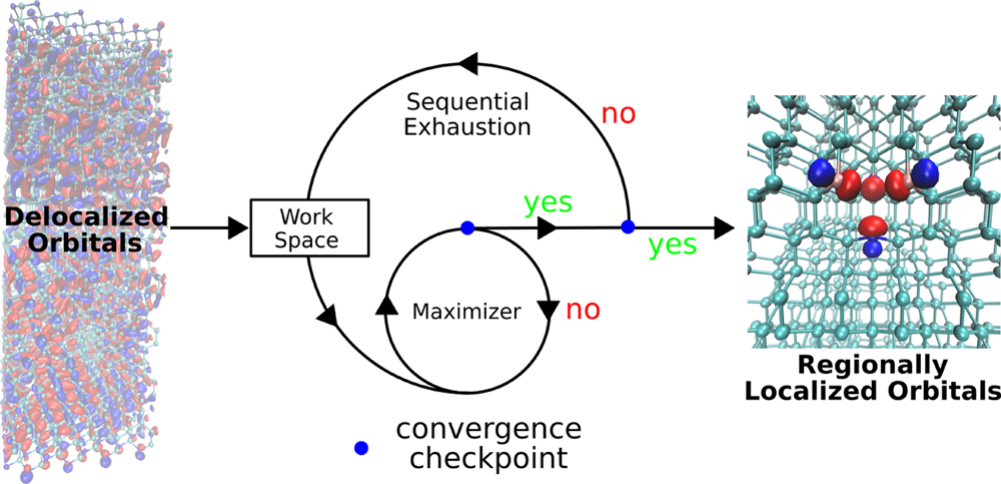

Wannier functions have become a powerful tool in the electronic structure calculations of extended systems. The generalized Pipek-Mezey Wannier functions exhibit appealing characteristics (e.g., reaching an optimal localization and the separation of the σ–π orbitals) compared with other schemes. However, when applied to giant nanoscale systems, the orbital localization suffers from a large computational cost overhead if one is interested in localized states in a small fragment of the system. Herein, we present a swift, efficient, and robust approach for obtaining regionally localized orbitals of a subsystem within the generalized Pipek-Mezey scheme. The proposed algorithm introduces a reduced work space and sequentially exhausts the entire orbital space until the convergence of the localization functional. It tackles systems with ∼10000 electrons within 0.5 h with no loss in localization quality compared to the traditional approach. Regionally localized orbitals with a higher extent of localization are obtained via judiciously extending the subsystem’s size. Exemplifying on large bulk and a 4 nm wide slab of diamond with an NV^–^ center, we demonstrate the methodology and discuss how the choice of the localization region affects the excitation energy of the defect. Furthermore, we show how the sequential algorithm is easily extended to stochastic methodologies that do not provide individual single-particle eigenstates. It is thus a promising tool to obtain regionally localized states for solving the electronic structure problems of a subsystem embedded in giant condensed systems.

## Introduction

Localized orbitals are widely used in electronic structure computations for multiple purposes: conceptually, they can provide valuable information about chemical bonding and chemical properties of molecules and materials. More importantly, they allow the evaluation of nonlocal two-body interaction integrals at a significantly reduced cost due to the reduced spatial overlaps. Hence, they represent a powerful tool in mean-field and postmean-field electronic structure calculations such as hybrid functional calculations,^1,2^ density functional theory with the Hubbard correction term,^3,4^ or many-body calculations.^5,6^ In the same vein, the maximally localized orbital descriptions are optimal for treating correlation phenomena since (due to the locality) the number of “inter-site” interactions is minimal, and the effective size of the problem is smaller. As a result, optimally localized states are essential in the context of embedding and downfolding for many-electron problems.^7−10^

Orbital localization approaches can be categorized by whether a cost function is optimized or not. The selected columns of the density matrix (SCDM)^11^ method and projection with a minimal atomic basis^10,12^ are representative localization schemes without optimizing a cost function. Within the optimization techniques, several functionals have been proposed: the Foster-Boys (FB) scheme^13−15^ minimizes the spatial extension of the orbitals and leads to maximally localized Wannier functions (MLWF)^16,17^ in periodic solids, while the Edmiston-Ruedenberg (ER) approach^15,18,19^ maximizes the self-repulsion energy. von Niessen^20^ introduced another functional that maximizes the charge-density overlap. Pipek-Mezey (PM)^21^ proposed to minimize the mean delocalization measure (defined later). Arguably, the most popular approaches are the FB scheme for molecules and the MLWF for periodic solids due to their  scaling (*N* is the number of electrons), but these schemes suffer from the mixture of σ – π bonds, commonly known as “banana” orbitals.^21,22^ The ER approach provides more localized orbitals than the FB and supports the σ – π separation. However, its computational cost scales as steeply as , preventing it from practical applications in large systems.

Among these functional-optimization approaches, PM localization is the most appealing approach. It can provide high spatial localization and the separation of σ – π characters of chemical bonds compared with the FB counterpart. At the same time, the scaling of PM localization is  only, i.e., significantly lower than the ER counterpart. Because of the mathematically ill-defined Mulliken charges^23^ in the original scheme, the PM localization has been generalized to various partial charge schemes.^24,25^ The generalized PM approach is robust with respect to the choice of the partial charge.^25^ Recently, the PM localized molecular orbital formalism has been further expanded to periodic systems.^22^ This generalized Pipek-Mezey Wannier Functions (G-PMWF) approach retains the advantages (particularly stronger localization) compared with MLWF.

The iterative optimization with  scaling per iteration, however, still translates to a relatively high computational cost and requires that all single-particle states are known. This becomes a bottleneck for giant systems: the overhead is substantial when one is interested only in a small fraction of the system, such as maximally localized orbitals associated with a point defect in solids, an adsorbate molecule on a surface, or molecular states in a complex environment. Here, handling the entire problem is often necessary, despite only a fraction of localized states being sought. Such nanoscale problems involve thousands of electrons. To generate PMWFs or localized orbitals with comparable quality, the prevalent strategy is to lower the number of iteration steps necessary to reach the optimum, e.g., by a robust solver.^26,27^ Although the proposed scheme is either iteration-free^26^ or can effectively lower the iteration steps toward convergence,^27^ an auxiliary set of functions or atomic basis is still required in the localization process. The computational scaling to the system’s size is not seen improved either. Further, for truly large systems with thousands of electrons, one would employ techniques that avoid the use (or knowledge) of all single-particle states.^28−45^

Herein, we present a new and complementary top-down approach leading to a fast, efficient, and robust orbital localization algorithm via sequentially exhausting the entire orbital space. It is beneficial for obtaining regionally localized orbitals for a subsystem within the G-PMWF scheme. In contrast to other methods, the problem’s dimensionality is reduced from the outset by partitioning the orbital space. As our work space is effectively compressed, the dimensionality of the relevant matrices in the G-PMWF scheme is much smaller, and therefore, the time per iteration step is shortened by orders of magnitude. The unitary transform is performed iteratively until convergence. The transformation starts directly either with (i) the canonical real-space delocalized orbitals without any external or auxiliary atomic basis set^26,27,46^ or (ii) an initial guess of the subspace of localized single-particle orbitals (which can be obtained by, e.g., filtering^28,30,31,33,41,43^). The compression of dimensionality helps to reduce the scaling of the method with the number of electrons to be linear. The completeness of sequentially exhausting the orbital space is demonstrated by the converged localization functional. We test the quality of the localized basis by constructing an effective Hubbard model for the negatively charged nitrogen-vacancy (NV^–^) defect center in diamond and computing its optical transition energies in bulk supercells and a large (4 nm thick) slab containing nearly 10,000 electrons. Excellent agreement between the sequential exhausting approach and the full space approach is achieved for the computation of optical transition energies. The accuracy of Hubbard model calculations is further improved by the Wannier function basis obtained from the subsystem with an extended size. In the last section, we provide a thorough discussion of how the choice of localization affects the excitation energies of the embedded NV^–^ center.

## Theory

### Generalized Pipek-Mezey Wannier Functions

In this subsection, we briefly revisit the G-PMWF formalism^22^ to clarify the motivation for this work. The G-PMWF seeks to minimize the mean delocalization measure  defined as^21^
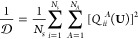
1where *i* denotes the *i*^th^ state, and *N*_*s*_ represents the number of states that spans a particular orbital space. *A* is the *A*^th^ atom in the system, and *N*_*A*_ is the number of atoms in the system. *Q* is termed the atomic partial charge matrix (defined below). In practice,  represents the partial charge on atom *A* contributed by state *i*. **U** is the unitary matrix that transforms the orbitals. Minimizing  is equivalent to maximizing the following functional 
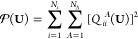
2

The stationary point of  corresponds to the unitary matrix **U** that transforms the canonical states into Pipek-Mezey localized states
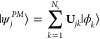
3where |ϕ_*k*_⟩ represents the canonical state.

Generally, the value of  is iteratively maximized until reaching convergence. In the *n*^th^ iteration step, the *Q* matrix can be calculated by
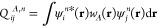
4Here, represents either the transformed state (*n* > 0) or the canonical state (*n* = 0). In the G-PMWF formalism, *w*_*A*_ denotes the atomic weight function using real-space partitioning,^22,25^ e.g., Gaussian weight.^47^

For *n* ≥ 1, the *Q* matrix can also be transformed by
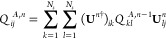
5

Note that in practice, the *Q* matrix has a dimensionality of . The number of elements reaches 10^9^ for a system with 10^3^ atoms and 10^3^ occupied states. Furthermore, in our real-space implementation, the theoretical scaling of the method is , where *N*_*g*_ denotes the number of grid points in real space. Our numerical results for the defect center in diamond are close to this theoretical behavior, as discussed in the Results and Discussion section.

### Fragmentation and Sequential Variant of G-PMWF

This subsection presents an efficient algorithm to obtain a subset of PMWFs localized on a specific set of atoms.

#### Fragmentation Treatment

Conventionally, one has to localize all *N*_*s*_ states and then identify *N*_*rl*_ states that are regionally localized on the selected atoms. For instance, for a CH_4_ molecule surrounded by other atoms/molecules, *N*_*rl*_ will be four if considering only the valence electrons and doubly occupancy. When *N*_*rl*_ ≪ *N*_*s*_, this approach suffers from a significant overhead. This is quite limiting when nanoscale systems are considered: the dimensionality of matrix *Q* and the computational scaling make it challenging to work with thousands of electrons. Previously, we introduced a modified form of the PM functional to account for  selected atoms only and search for the *N*_*rl*_ states directly.^6^ Such a modification is equivalent to the search of a local maximum of  on the selected atoms, and it reduces the dimensionality to . In this work, we further compress the  to simply 1 by creating a single fragment from the subset of atoms. Unlike the “fragment” proposed in the FB scheme,^46^ our definition of a fragment uses the atomic weight function *w*_*A*_
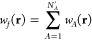
6where *f* denotes the fragment of interest. The localization functional thus becomes
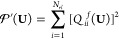
7where  is the modified PM functional for the fragment.

Note that (i) the unitary transform is still performed on *all N*_*s*_ states that need to be known, and (ii) the *N*_*rl*_ states are identified from *N*_*s*_ by evaluating the partial charge on the selected fragment. In this context, we define the measure of the locality of a specific state on the fragment as
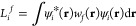
8Its value ranges from 0 (not localized) to 1 (most localized). Only the top *N*_*rl*_ states of the *N*_*s*_ states in the decreasing order of  are considered the regionally localized Wannier functions on the fragment. In the following text, we denote this fragmentation variant of G-PMWF as “F-PMWF”.

Next, the F-PMWF approach is broken into two steps: (1) maximize  (eq 7) and find the *N*_*rl*_ states that are localized on the fragment and (2) maximize the canonical  defined as
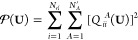
using the *N*_*rl*_ states from step 1 and obtain localized states on each individual atom of the fragment.

Essentially, the first step is a “folding” step where the electron density is effectively localized on the fragment disregarding the individual atoms. The second step is instead an “unfolding” step where the electronic states obtained from step 1 are unfolded onto each individual atom in the fragment.

The *Q* matrix is reduced to  in step 1 and to  in step 2, respectively. The second step is trivial in cost since *N*_*rl*_ is often much smaller than *N*_*s*_. However, the first step can still be expensive when working with thousands of electrons, and the knowledge of *N*_*s*_ eigenstates is necessary.

#### Sequential Exhausting of the Full Orbital Space

To further compress the *N*_*s*_ in the maximization process and, in principle, avoid the knowledge of *N*_*s*_ states altogether, we introduce a *sequential variant* of F-PMWF, sF-PMWF. We first review the approach which assumes *N*_*s*_ states are available, and at the end of this section, we extend it to a more generalized case when the eigenstates do not need to be known *a priori*.

**Figure 1:**
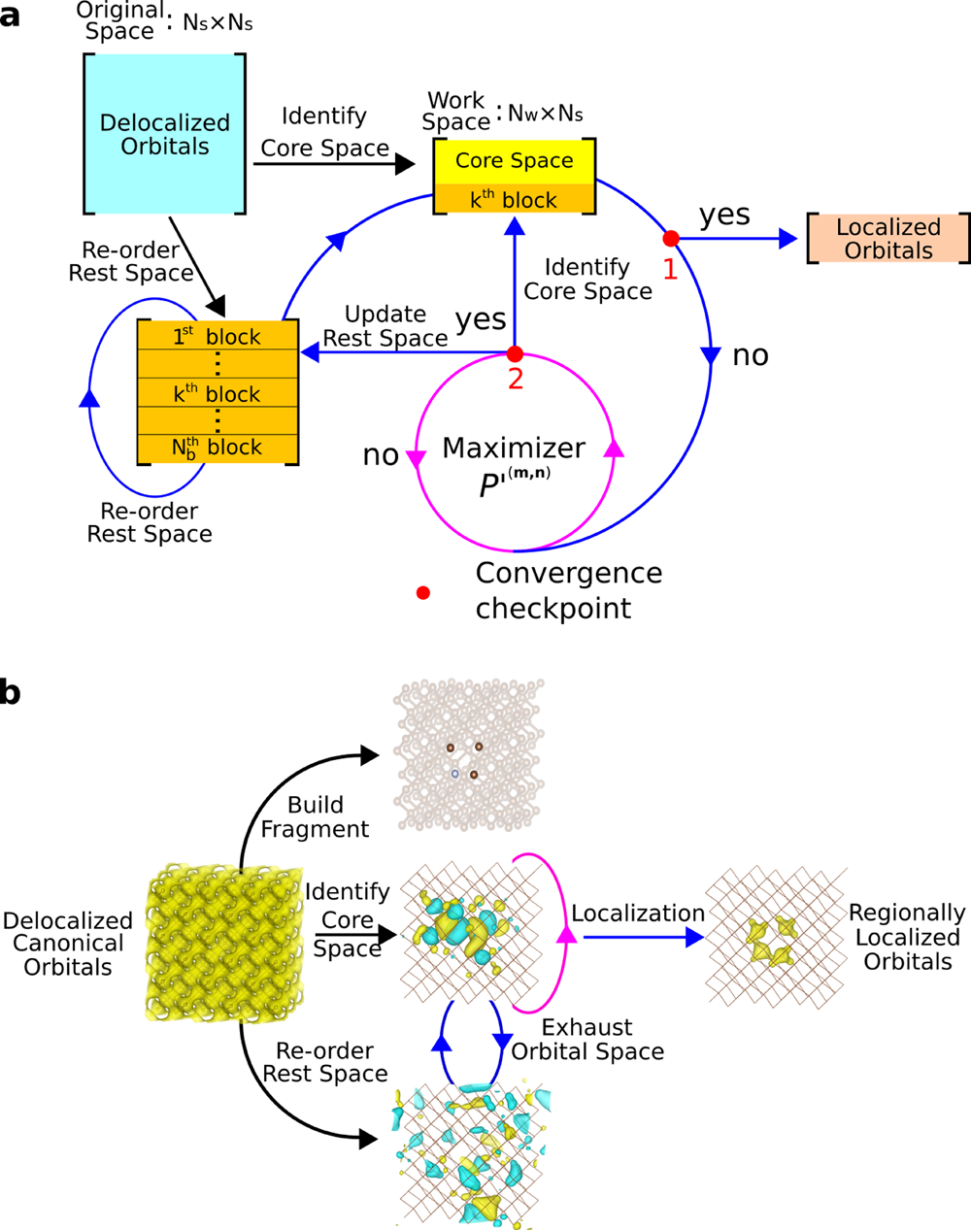
(a) Schematic illustration of the sF-PMWF method. Each row of the matrix represents a single-particle state in the canonical |ϕ_*j*_⟩ basis. *N*_*s*_ represents the number of states that define the original space, while *N*_*w*_ represents the number of states in the actual work space. *P*′ is the modified PM objective functional. The index *m* denotes the iterative step of the outer-loop (blue). The index *n* denotes the iterative step of the inner-loop (magenta). (b) sF-PMWF method exemplified on the NV^–^ center in diamond. The electron density represents the occupied space consisting of *N*_*s*_ delocalized canonical orbitals. The fragment is built with four selected atoms. The core space is first defined by *N*_*c*_ relatively localized canonical states and then sequentially localized on the selected fragment. The rest space is represented by *N*_*s*_ – *N*_*c*_ delocalized states over the whole system. The output is a set of regionally localized Wannier functions on the selected fragment. The isosurface value is set at 0.1 for the electron density and 0.05 for the single-paricle orbital.

The sF-PMWF approach incorporates an additional iterative loop (“outer-loop”) to maximize the functional  successively. The idea is schematically presented in Figure 1a. A generalized original (entire) space, either occupied or unoccupied, is spanned by *N*_*s*_ orthonormal canonical states. The initial matrix that contains the canonical states is the identity matrix, and each row of the matrix contains the coefficients of a single-particle state in the canonical basis. The number of rows represents the number of states used in the *Q* matrix. The black lines and arrows stand for the initialization of the localization procedure. The outer-loop is guided by the blue lines and arrows, while the magenta lines and arrows guide the inner-loop (maximizer). The red points denote the convergence checkpoints.

Our goal is to find only *N*_*rl*_ states that are spatially localized on a selected fragment. We seek to minimize the cost of the calculation by neglecting the localization in the other regions of the systems. The general procedure is as follows:

First, we assume that in practical calculations, it may be necessary to account for a “buffer”, i.e., we search for *N*_*c*_ ≥ *N*_*rl*_ states (where *N*_*c*_ is typically similar to *N*_*rl*_ in magnitude). We denote the *N*_*c*_ most localized orbitals chosen based on the value of  (eq 8) as “core states”, and the “core space” is spanned by such *N*_*c*_ states. The original space is essentially split into two, the core and its complement (denoted “rest space”). The states in the rest space are then reordered upon their locality (eq 8) for the next step.

Second, a work space is built with a dimensionality of *N*_*w*_ × *N*_*s*_, where *N*_*c*_ < *N*_*w*_ ≪ *N*_*s*_. The first part of the work space is filled by the core states (the yellow region). On the other hand, the rest space is partitioned into *N*_*b*_ blocks according to the value of *N*_*r*_, which is an arbitrary integer parameter (1 ) that denotes the number of states from the rest space, and note that the states in the rest space have been reordered in the decreasing order of . The number of states in each block satisfies the following equations
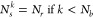
9and
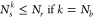
10Here, represents the actual number of states in the *k*^th^ block. The rest space is sequentially updated (explained in the next step) and can be reaccessed during the localization process. The index *m* denotes the *m*^th^ iteration step in the outer-loop, and the *m* (*m* > 0) and *k* are connected by
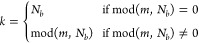
11Here, we define one “macro-cycle” when the outer-loop exhausts all the blocks in the rest space once, i.e., the full orbital space is transformed once.

Third, the initial (*m* = 0) objective functional value (eq 7) is calculated for the work space, and the change of the PM functional in the outer-loop is defined as
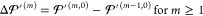
12The convergence checkpoint 1 in Figure 1a evaluates the  as well as the accumulative step *m*. The iteration will exit the outer-loop if either
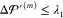
13or
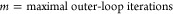
14is satisfied. Here, λ_1_ is a convergence threshold. The λ_1_ value and the maximal outer-loop iterations are carefully chosen to converge the localization (see the next section). If the iteration does not exit the loop, the index *m* will become *m* + 1, and the corresponding *k*^th^ (eq 11) block will fill the second part of the work space. The constructed work space then enters the maximization solver (the inner-loop in magenta). The change of the PM functional in the inner-loop is defined as
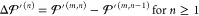
15Here, *n* denotes the iteration step (if iterative maximization is needed) in the inner-loop. The convergence checkpoint 2 evaluates the  as well as the accumulative step *n*. The iteration will exit the inner-loop if either
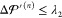
16or
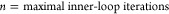
17is satisfied. Here, λ_2_ is another convergence threshold. The λ_2_ value and the maximal inner-loop iterations are carefully chosen to allow the work space to reach the maximum smoothly (see the next section). Once exiting, the core space is identified from the transformed work space, and the residues of the work space replace the  states in the *k*^th^ block. This operation is denoted as “the update of the rest space” since both the core and rest spaces are dynamic during the maximization. The index *n* is reset to 0, and the  arrives at the convergence checkpoint 1. If the iteration does not exit the loop, the next block then fills the work space to re-enter the maximizer. With all the *N*_*b*_ blocks exhausted and updated, the states in the rest space will be reordered again for the next macro-cycle.

In Figure 1b, we provide a concrete example where the sF-PMWF algorithm is applied to search for *N*_*rl*_ = 16 regionally localized Wannier functions on the NV^–^ center in diamond. The original space is the occupied space consisting of *N*_*s*_ = 432 delocalized canonical orbitals, represented by the electron density. The fragment is built with the four atoms at the NV^–^ center, and then *N*_*c*_ ≥ *N*_*rl*_ relatively localized (based on eq 8) canonical states are identified from the original space to form the core space. The complementary *N*_*s*_ – *N*_*c*_ states are reordered and form the rest space. The rest space is then sequentially exhausted and updated at a step of *N*_*r*_ until convergence. The output is *N*_*rl*_ Wannier functions that are regionally localized on the selected fragment (represented by the electron density).

In practice, the outer-loop (identify the core space, construct the work space, maximization, and update the rest space) has to be iterated multiple times until the  is converged. In general, each iteration step in the outer-loop feeds the core space with the ingredients to localize itself and sequentially exhaust the full orbital space until convergence. However, the cost of the calculation depends primarily on the size of the work space *N*_*w*_. A small *N*_*r*_ might require extra outer-loop iterations, but the cost of each maximization (“inner-loop”) should be orders of magnitude smaller than the traditional full-space approach.

So far, we have assumed that a basis of individual single-particle states is known (e.g., obtained by a deterministic DFT calculation). However, this procedure is trivially extended even to other cases, e.g., when stochastic DFT is employed.^28−33^ For simplicity (and without loss of generality), we assume the localization is performed in the occupied subspace. Here, the sF-PMWF calculation is initialized by constructing a guess of *N*_*c*_ random vectors |ζ⟩, which are projected onto the occupied subspace as . These *N*_*c*_ random states then enter the core space in Figure 1a. Here, the projector  is a low-pass filter constructed from the Fermi operator leveraging the knowledge of the chemical potential.^28−33,41,43^ Next, in each outer-loop step, one creates a block of random vectors , which have to be mutually orthogonal as well as orthogonal to the *N*_*c*_ core states via, e.g., the Gram-Schmidt process. Here, *r* denotes the rest space, and *m* denotes the *m*^th^ step in the outer-loop. This block of random states follows the procedure in Figure 1a to fill the work space. Note that this block of random vectors represents the entire orthogonal complement to the core space.

Combined with the fragmentation treatment, the number of elements in *Q* is reduced from  to , and the unitary matrices are also reduced from  to . Such a reduction in dimensionality is expected to shorten the time spent on each iteration step as long as *N*_*w*_ ≪ *N*_*s*_. The cost of the stochastic method (which does not require the knowledge of the *N*_*s*_ eigenstates) is higher due to the additional orthogonalization process. In the Results and Discussion section, we show that the total wall time spent on a job becomes much shorter, especially for large systems, at the expense of more inner-loop steps. Most importantly, the localized states obtained from sF-PMWF are practically identical to those obtained from the traditional F-PMWF approach.

## Computational Details

### F-PMWF and sF-PMWF

A shared memory approach is employed to parallelize the do-loops (via OpenMP). Several real-space partitioning schemes^47−51^ for the atomic weight function in eq 4 have been tested within the PM localization framework.^25^ It turns out the resulting localized orbitals are insensitive^22,25^ to its choice. This robustness of the G-PMWF approach allows choosing the weight function for computational convenience;^22,25^ in this work, Hirshfeld partitioning^47^ is used to calculate the *Q* matrix in eq 4. The actual implementation can be found in ref (22). For simplicity, we employ the steepest ascent (SA) algorithm^52−54^ to maximize the PM functional  and . Note that other extremization procedures will likely further reduce the cost of the inner-loop, but they do not have a decisive effect on the overall scaling. The ascending step is set at 5.0 in the beginning and divided by 1.1 each time the change of PM functional  appears negative. In calculations using a stochastic basis, the random states are constructed using Fortran random number generator. The random number generator employs seeds that change in each outer-loop step. These random states are then orthogonalized by the Gram-Schmidt process detailed in the Supporting Information (SI).

In F-PMWF calculations, the λ_2_ is set at 5 × 10^–7^, and it has to be consecutively hit three times to ensure smooth convergence. In sF-PMWF calculations, the λ_2_ is set at 1 × 10^–7^ in the inner-loop, which also has to be hit three times consecutively. The λ_1_ is set at 5 × 10^–7^ for the outer-loop. The maximal iteration step is set at 2000 for *n* and 5000 for *m*.

To avoid the spurious convergence or local maximum issue, a special criterion is devised for the sF-PMWF. The principle comes from the full-space F-PMWF. When the core space reaches the maximum localization, the whole rest space should no longer increase the  by , and neither should a subspace in the rest space contribute further; and thus, the  of each block in one complete macro-cycle is evaluated simultaneously. Only if the maximal  satisfies the criterion  will the  be considered converged. This also means that once the first block re-enters the work space, all the blocks must be exhausted to decide the convergence. This might lead to a slight increase in cost but guarantees that the sF-PMWF reaches the convergence in the same manner as the F-PMWF.

The sF-PMWF calculation can be easily restarted as long as one keeps the checkpoint file at the *m*^th^ step and sets the outer-loop to start with *m* + 1. The source code is posted on git-hub and available for download.

### Model Systems

As a test case, we investigate the NV^–^ center in 3D periodic diamond supercells and a 2D slab. The relaxed chemical structures of the investigated systems are provided in Figure S1. The atomic relaxations of the NV^–^ defect center in 3D periodic diamond supercells with 215, 511, and 999 atoms are performed using the QuantumESPRESSO package^55^ employing the Tkatchenko-Scheffler’s total energy corrections.^56^ For the 111 nitrogen terminated surface slab 2D periodic calculations, the surface relaxation also employs the Effective Screening Medium correction.^57^ The atom relaxation of the surface terminated with nitrogen atoms is performed on a smaller slab with 24 atoms, which corresponds to the 1 × 1 × 2 supercell. The relaxed top and bottom surfaces were then substituted into a large 4 × 4 × 6 (1.5 × 1.7 × 4.7 nm) supercell containing 2303 atoms. The 111 surface is set normal to the *z*-direction. The relaxed structure of the NV^–^ center is cut out from a 511-atom supercell in a way that the N–V axis is normal to the 111 surface. This supercell is then substituted in the middle of the 111 nitrogen terminated surface 4 × 4 × 6 slab at the 2 nm depth from the surface.

The starting-point calculations for all systems are performed with a real-space DFT implementation, employing regular grids, Troullier-Martins pseudopotentials,^58^ and the PBE^59^ exchange-correlation functional. For 3D periodic structures, we use a kinetic energy cutoff of 26 hartree to converge the eigenvalue variation to <5 meV. The real-space grids of 68 × 68 × 68, 92 × 92 × 92, and 112 × 112 × 112 with the spacing of 0.3 *a*_0_ are used for 215-atom, 511-atom, and 999-atom supercells, respectively. The grid of 70 × 82 × 338 with the spacing of 0.4 *a*_0_ is used for the 2303-atom slab supercell. The generated canonical Kohn–Sham eigenstates are used for the subsequent orbital localization.

## Results and Discussion

The full-space F-PMWF and the proposed sF-PMWF methods are applied to obtain regionally localized states on the NV^–^ center in diamond. The NV^–^ center is composed of three carbon atoms and one nitrogen atom that are mutually nonbonded. The fragment in the actual calculations is constructed with these four atoms (see Figure 1b) unless stated otherwise. The number of regionally localized states, *N*_*rl*_, is 16 on the constructed fragment. Two types of systems, solids and slab, are studied. For the solids, three supercells of different sizes are investigated. The number of occupied states, *N*_*s*_, for each system is 432, 1024, and 2000, respectively. For the slab, the regionally localized states are identified from a supercell with 2303 atoms and 4656 occupied states.

### Completeness of sF-PMWF

First, we investigate the completeness of the sequential exhausting approach, i.e., whether the sF-PMWF can reproduce the same results as the F-PMWF. To contrast the sF-PMWF method, we perform F-PMWF localization on the 511-atom system using a truncated orbital space. This is a common technique to lower the cost by filtering out a portion of canonical states upon the eigenenergy (eigenvalue). Only eigenstates within a specific energy range (termed as the “energy window”) are selected for localization. We tested two energy windows (10 and 20 eV below the Fermi level, respectively) on obtaining the localized Wannier function basis. Upon visual inspection, the results do not look too different, but when applied to compute the optical transitions in the NV^–^ center (see “Excited states of the NV^–^ center” in the SI), we see considerable differences in the energies (Table S1). The results from the truncated space are highly underestimated compared with the results from the full space. The energy-windowing technique fails since, to reach optimal localization, the maximum possible Bloch states are needed to be transformed, i.e., *all* the occupied states are necessary. To localize electronic states on a selected fragment, choosing states with significant spatial distribution on the fragment is more critical than the choice of the energy window for the F-PMWF technique. The degree of localization critically depends on what fraction of states that overlap with the selected fragment is included. Note that this is not necessarily related to the energy of the corresponding canonical mean-field state or the size of the energy window, i.e., even states energetically far from the defect state can be important and may plague the frozen window approach. The proposed sF-PMWF method does not have this issue, and we demonstrate its completeness below.

**Figure 2:**
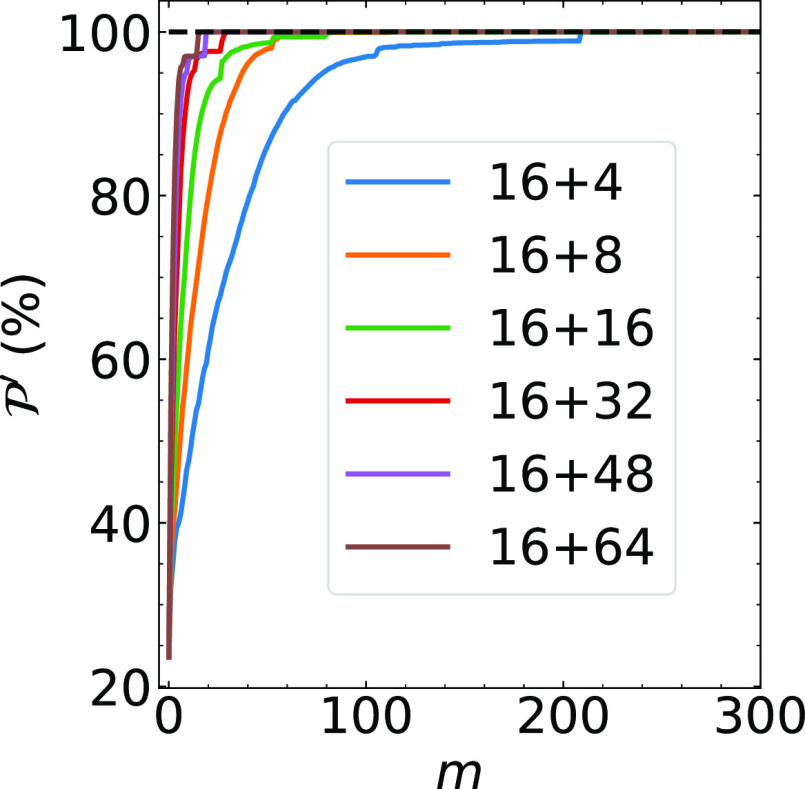
Convergence of the functional  with respect to the outer-loop step *m* for the NV^–^ center of the 215-atom system. Each curve is labeled by the combination of *N*_*c*_ and *N*_*r*_.

**Figure 3:**
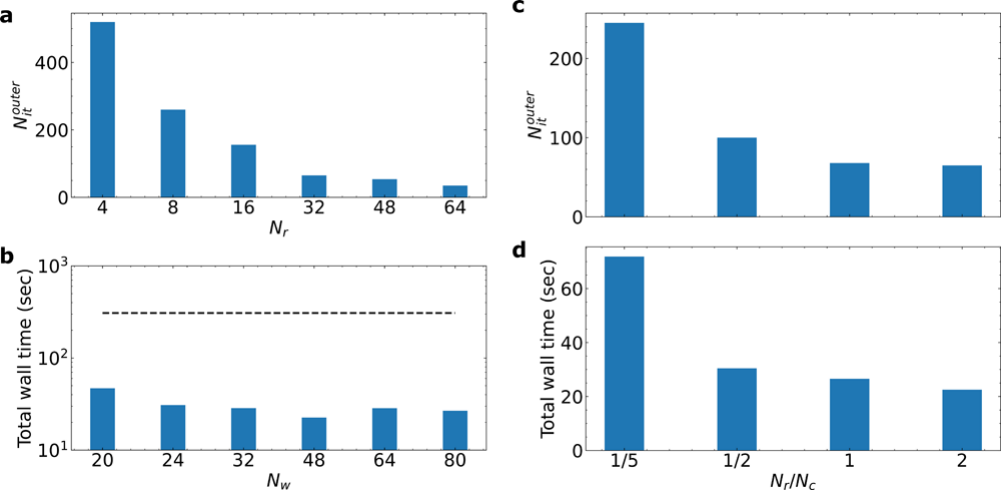
Left: Investigation of different combinations of *N*_*c*_ and *N*_*r*_ for the localization on the NV^–^ center of the 215-atom cell. *N*_*c*_ is fixed at 16. (a) Number of iteration steps in the outer-loop as a function of the *N*_*r*_. (b) Total wall time of the calculation as a function of *N*_*w*_. The dashed line indicates the total wall time from the F-PMWF method using the full orbital space. Right: Investigation of different combinations of *N*_*c*_ and *N*_*r*_ for the localization on the NV^–^ center of the 215-atom cell. *N*_*w*_ is fixed at 48. (c) Number of iteration steps in the outer-loop as a function of the *N*_*r*_/*N*_*c*_ ratio. (d) The total wall time as a function of the *N*_*r*_/*N*_*c*_ ratio.

We first illustrate the completeness in detail using the 215-atom system. To initialize the sF-PMWF calculations, the *N*_*c*_ parameter takes 16 (minimum), i.e., we take no “buffer”. For convenience, we only consider combinations with *N*_*r*_ being an integer multiple of *N*_*c*_ and vice versa. Several *N*_*r*_ ranging from 4 to 64 are tested. Figure 2 shows the maximized , which measures the degree of localization (eq 7) relative to the converged maximized value using the full space , as a function of the accumulative outer-loop step *m*. It can be clearly seen that 100% of the  is sequentially recovered regardless of the (*N*_*c*_, *N*_*r*_) combination. The maximization of each curve presented in Figure 2 is not smooth, i.e., spikes are observed at the step where the iteration enters a new macro-cycle. In fact, at least 94% of the converged  has been gained after the first macro-cycle (see Table S2). As the *N*_*r*_ increases, fewer and fewer iteration steps  are required to reach convergence (Figure 3a), and theoretically, the  should be reduced to two (the second step is to exit the outer-loop) if one takes *N*_*r*_ = *N*_*s*_ – *N*_*c*_ to work directly in the full space. However, the reduction in  does not necessarily lead to a shorter job time. Note that the time per outer-loop iteration (*t*^*outer*^) increases with a scaling of  (see Figure S3) for the 215-atom system. Figure 3b shows the total wall time of each job as a function of the *N*_*w*_ with *N*_*c*_ fixed at 16. The  dominates the total wall time when *N*_*w*_ is small (48). In this regime, reducing the number of iterations lowers the total wall time effectively. When the *N*_*w*_ is larger, however, the *t*^*outer*^ becomes the dominating factor, and the total wall time increases even though the  decreases. The trade-off between  and *t*^*outer*^ suggests there exists an optimal combination of *N*_*c*_ and *N*_*r*_ for a specific system to minimize the total cost.

We also test the sF-PMWF calculation employing a set of stochastic basis that represents the rest space. The same parameter combination (16,32) is used. The 16 core states are taken directly from the canonical eigenstates based on the locality, while the 32 stochastic states are constructed in a three-step manner (see “Preparation of stochastic basis” in the SI). Compared with the (16,32) calculation using the deterministic basis, the stochastic approach exhibits the same completeness in exhausting the full orbital space, as seen from the converged  and . Nevertheless, more outer-loop iterations are needed due to the randomized search, and the time per iteration also becomes longer (3.47 s versus 0.32 s) due to the Gram-Schmidt orthogonalization process; therefore, the total wall time increases to 729 s (see the last row in Table S2). For the evolution of the objective functional in comparison with the deterministic counterpart, the stochastic approach converges more smoothly (see Figure S4). The stochastic basis search does not show competitive efficiency versus the full-space approach (308 s) for such a small system. In the following section, we show the stochastic basis approach becomes more efficient than the full-space counterpart for a larger system. However, we emphasize that the advantage of sF-PMWF does not hinge on this stochastic extension but enables it. In most of our results, we will focus on the fully deterministic approach in which the knowledge of *N*_*s*_ states is assumed.

The behavior of the sF-PMWF method discussed above is also observed for the 511-atom system (Figure S5 and Table S3 in the SI), confirming the generality of the completeness.

### Optimization of Work Space

In the previous section, we observe a trade-off between  and *t*^*outer*^, which implies a possibly optimal parameter combination. To further understand the choices of *N*_*c*_ and *N*_*r*_, several other combinations with *N*_*c*_ > 16 are tested on the 215-atom system. The maximal  and  are secured regardless of the (*N*_*c*_, *N*_*r*_) combination, indicating that the convergence of  is insensitive to the choices of these two parameters. For *N*_*c*_ fixed at 16, the time-to-solution reaches a minimum when *N*_*w*_ = 48, as shown in Figure 3b. For *N*_*w*_ fixed at 48, different ratios of *N*_*r*_/*N*_*c*_ are tested. The results suggest that the larger the *N*_*r*_, the smaller the  (Figure 3c). Note that the *t*^*outer*^ depends solely on the *N*_*w*_ (Table S2), and therefore, a smaller  translates directly to a shorter wall time (Figure 3d). The numerical results are summarized in Table S2. This behavior is further observed in the 511-atom system (see Figure S6).

To further quantify our observations above, we examine the time per macro-cycle (*t*^*macro*^) and the number of macro-cycles (*n*^*macro*^) shown in Table 1. The variation of the total wall time (*t*^*tot*^) agrees well with the *t*^*macro*^ among different (*N*_*c*_, *N*_*r*_) combinations since the *n*^*macro*^ in each trial does not differ too much from one another (*n*^*macro*^ = 5 ± 1). The total wall time is essentially very close to *n*^*macro*^ × *t*^*macro*^. The scaling of *t*^*macro*^, in our sF-PMWF algorithm, can be approximately expressed as
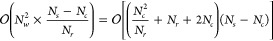
18With *N*_*s*_ and *N*_*c*_ fixed, the right-hand side (RHS) of eq 18 is a function of *N*_*r*_ with a theoretical minimum for some nonzero *N*_*r*_, and thus, eq 18 explains the existence of an optimal (*N*_*c*_, *N*_*r*_) combination as observed. We note that the RHS of eq 18 is also crucial in explaining the scaling of our sF-PMWF method with respect to *N*_*s*_ as discussed in the following section.

**Table 1:** Timing Data of Orbital Localization Performed on the 215-Atom System Using sF-PMWF

(*N_c_*, *N_r_*)	*t*^*tot*^ (s)	*t*^*macro*^ (s)	*n*^*macro*^
(16,4)	47	9.07	5
(16,8)	31	5.87	5
(16,16)	29	4.65	6
(16,32)	22	4.19	5
(16,48)	28	4.51	6
(16,64)	27	5.09	5

To conclude, the “buffer” seems to be unnecessary for the core space, i.e., *N*_*c*_ can be set directly as *N*_*rl*_ for a specific fragment. The work space optimization then depends solely on the choice of *N*_*r*_, and there exists an optimal *N*_*r*_. Nevertheless, the cost of the investigated sF-PMWF calculations without optimization is already absolutely lower than that of F-PMWF regardless of the *N*_*r*_ (see Figure 3b and Figure S5b). The protocol of choosing *N*_*c*_ and *N*_*r*_ is suggested to be *N*_*c*_ = *N*_*rl*_ and *N*_*r*_ = 2*N*_*c*_ since it leads to a local minimum in the total wall time.

This protocol is then applied to the 999-atom system, and two additional combinations of *N*_*c*_ and *N*_*r*_ are also tested. The (16,32) combination still leads to a cost minimum and is 85 times faster than the F-PMWF (see Table S4). Further, we also test the stochastic basis search with the 999-atom employing the (16,32) combination. The completeness of the stochastic exhausting is again confirmed by the converged  and . Although the stochastic approach is still more costly than the deterministic sequential counterpart, it is more efficient than F-PMWF (by roughly 50%) when applied to this system with ∼4000 electrons (see the last row of Table S4). Furthermore, ∼74% of the cost in the stochastic search comes from the Gram-Schmidt process, which advanced orthogonalization techniques can optimize. When combined with stochastic DFT, the total cost of orbital localization is expected to be much lower than the deterministic approach that requires the knowledge of the eigenstates in a system with tens of thousands of electrons.

For the 2303-atom system, the (16,32) combination successfully converges the  and produces localized states. Note that the cost can be lowered by 10% if the (16,48) combination is used, and if one searches further for the optimal *N*_*r*_ (or *N*_*w*_), it is possible to lower the cost further. However, for a fair comparison between one system and another, we use the timing from the (16,32) combination for the slab, which is already 412 times faster than the F-PMWF. The numerical results are provided in Table S5.

We also compare the time spent on folding and the unfolding steps, respectively (see Table S6). In each system, the cost of the unfolding step is merely 1–2% of the folding one since only *N*_*rl*_ states are transformed in the unfolding step, and thus, it is sufficient to evaluate just the cost of the folding step as the total cost of the orbital localization.

Finally, we remark that the (16,32) combination is stable and efficient for a given fragment regardless of the precise environment. This indicates that sF-PMWF is robust. Further, the consistent parameter combination clearly demonstrates the scaling of the sF-PMWF calculation with respect to the *N*_*s*_ as discussed in the next section.

### Scaling Analysis of sF-PMWF vs F-PMWF

To investigate the scaling of the sF-PMWF method, we normalize the timing data to the largest grid by
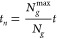
19where *t*_*n*_ represents the normalized time,  denotes the number of grid points of the largest system (the 2303-atom system), and *N*_*g*_ is the grid of each investigated system. The numeric data is summarized in Table 2. We report the results with a precision of 1 s for the total wall time and 0.01 s for the time per iteration/cycle. Here, we note that  represents the normalized total wall time,  and *n*^SA^ denote the normalized time per SA step and the number of SA steps in F-PMWF,  denotes the normalized time per outer-loop in sF-PMWF, and  and *n*^*macro*^ represent the normalized time per macro-cycle and number of macro-cycles in sF-PMWF.

**Table 2:** Normalized Timing Data of Orbtial Localization Performed on the Four Investigated Systems Using F-PMWF and sF-PMWF, Respectively

	F-PMWF			sF-PMWF			
system	*t*_*n*_^*tot*^ (s)	*t*_*n*_^SA^ (s)	*n*^SA^	*t*_*n*_^*tot*^ (s)	*t*_*n*_^*outer*^ (s)	*t*_*n*_^*macro*^ (s)	*n*^*macro*^
215-atom	1903	1.81	637	139	1.99	25.83	5
511-atom	18339	21.43	700	284	2.08	66.45	4
999-atom	58007	85.01	586	675	2.07	128.16	5
2303-atom	695370	1056.26	650	1683	1.89	266.02	6

**Figure 4:**
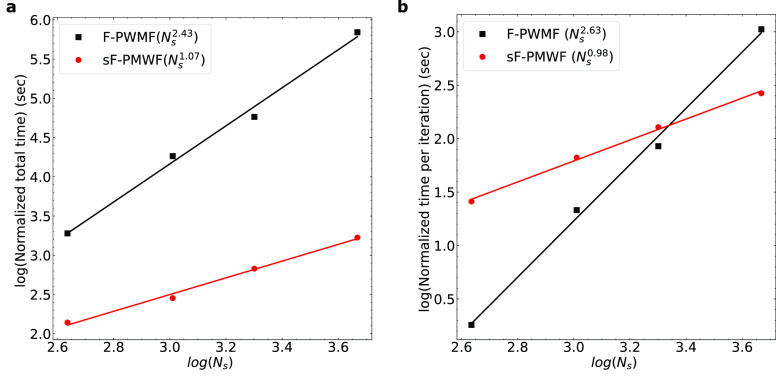
(a) The log of the normalized total job time plotted as a function of the log of *N*_*s*_. (b) The log of the normalized time per macro-cycle plotted as a function of the log of *N*_*s*_. The black line and square points represent results obtained from the F-PMWF method transforming the full orbital space at once. The red line and circle points represent results obtained from the sF-PMWF method sequentially transforming the orbital space. The scaling is derived from the slope of each fitting using the numeric data in Table 2.

In Figure 4a, the log of  is plotted as a function of the log of *N*_*s*_ for the four investigated systems. The scaling of the F-PMWF using the full orbital space is  (black line and square points). This is a bit higher than the theoretical  due to the other  do-loops, tasks related to parallelization, and practical executions (e.g., reading and writing of files). The sequential method, sF-PMWF, reduces the scaling from  to  (red line and circle points). This linear scaling is observed when the same protocol (16,32) applies to the four systems. Such an order of magnitude reduction in the scaling promises the efficiency of sF-PMWF when applied to much larger systems. In our largest system with 4656 states, the total wall time is shortened from 8 days to <0.5 h (Table 2, on a workstation with 2.5 GHz CPUs and parallelization on 60 cores).

The reduced scaling of sF-PMWF is largely attributed to the reduction of dimensionality during the maximization process. The efficiency is reflected mainly in the time per inner-loop iteration, *t*^*inner*^ in sF-PMWF and *t*^*SA*^ in F-PMWF. From 432 states to 4656 states, the  of the F-PMWF approach scales rapidly from 1.81 to 1056.26 s (Table 2). As shown in Figure 4b, the scaling of  in F-PMWF is . Further, Table 2 shows that the numbers of inner-loop iterations  in F-PMWF are reasonably large (600–700) and translate to a total scaling of  shown in Figure 4a.

In sF-PMWF, however, the *t*^*inner*^ remains constant and as low as  seconds regardless of the *N*_*s*_ (see Table S9). Although more SA iteration steps are required relative to the F-PMWF calculations (Figures S8 and S9), 1000 iterations now take as low as 0.5 s, and therefore, in sF-PMWF, the time spent in the maximizer is no more the dominating factor within an outer-loop step. It is more convenient to evaluate the efficiency of sF-PMWF by  and . We first study the scaling of the time per outer-loop step  with respect to the *N*_*s*_. It is shown that  hardly scales with respect to *N*_*s*_ when the same (*N*_*c*_, *N*_*r*_) combination is applied (see Figure S10). In addition,  scales almost linearly with *N*_*s*_ and gives a total scaling of .

A more direct derivation of linear-scaling is by evaluating  and *n*^*macro*^ summarized in Table 2. Interestingly, *n*^*macro*^ is very close between any two systems, being 5 ± 1. The total wall time is approximately the product of  and *n*^*macro*^; hence, it is sufficient to evaluate  only. In the previous section, eq 18 actually suggests that the scaling of *t*^*macro*^ depends linearly on *N*_*s*_. The normalized time per macro-cycle (, Table 2) is plotted as a function of *N*_*s*_ in Figure 4b. Note that  for F-PMWF coincides with  since the full orbital space is transformed at once in a single SA step. Here,we can clearly see the linear dependence  in sF-PMWF versus the  in F-PMWF. Although  for a specific system in sF-PMWF can be higher than that in F-PMWF, the evaluation of the number of macro-cycles, *n*^*macro*^ or *n*^*SA*^, is ∼5 for sF-PMW,F while it is ∼650 for the conventional F-PMWF. To conclude, eq 18 quantitatively explains the observed linear-scaling when the same (*N*_*c*_, *N*_*r*_) combination is applied to systems of different sizes.

### Localization Quality of sF-PMWF vs F-PMWF

#### Visualization of Localized Orbitals and Density

**Figure 5:**
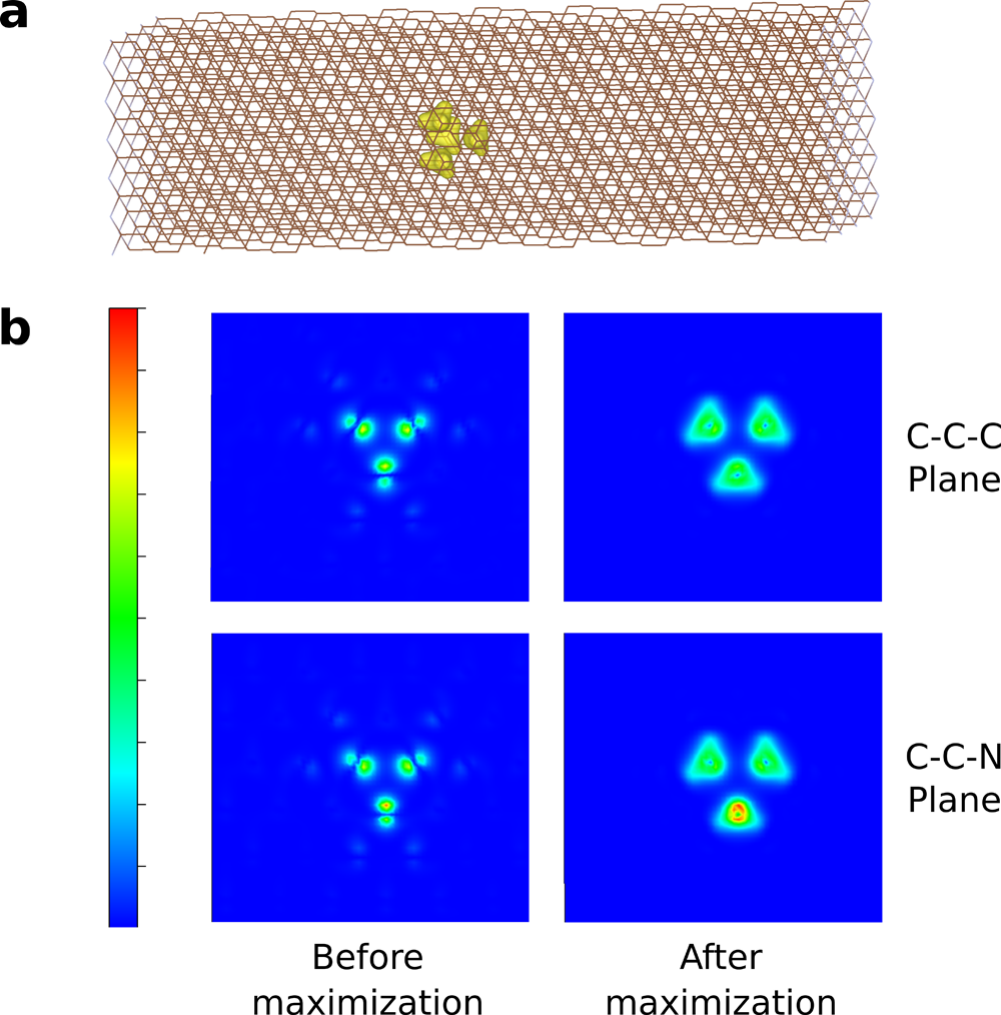
(a) Electron density constructed from the 16 regionally localized states around the NV^–^ center. The isosurface value is set at 0.05. (b) Density distribution sliced through the C–C–C plane (upper panels) and the C–C–N plane (lower panels) of the NV^–^ center in the slab. The left panels are constructed from the 16 most localized canonical states before the sF-PMWF calulation, and the right panels are constructed from the 16 regionally localized states after the sF-PMWF maximization.

In the previous section, the completeness of sF-PMWF has been demonstrated for the maximization of the modified PM functional  (eq 7). These 16 resulting states are localized on the fragment and serve as a subspace to further maximize the , which unfolds the states on each individual atom. The converged  and  between F-PMWF and sF-PMWF differ by no more than 0.0001 (<0.002%, see Tables S11 and S12) . Graphically, the 16 regional Wannier functions correspond to 9 C–C bonds, 3 C–N bonds, and 4 “p-like” states. The electron densities constructed from these 16 localized states are shown to be visually identical between the sF-PMWF and F-PMWF calculations (see Figures S11 to S13). The same agreement is also seen for the four selected individual “p-like” states (Figures S14 to S16) that are used in the following excited-state calculations. Figure 5a highlights the NV^–^ center in the slab using the regionally localized electron density. The obtained electron density conserves the spatial symmetry across the C–C–C plane and the C–C–N plane (Figure 5b). The left panels of Figure 5b show the electron density constructed from the 16 most localized canonical states, while the right panels present the maximized results from the sF-PMWF calculation. It can be clearly seen that electron density distribution becomes much more concentrated on the selected atoms, indicating the effectiveness of the localization.

To demonstrate that the sF-PMWF localization is subsystem-independent, an arbitrary carbon atom is chosen from each investigated system, and four regionally localized states are sought. The electron density around the selected C atom is successfully reproduced for each system (see Figure S17), confirming the generality of the sF-PMWF approach.

#### Excited States of the NV^–^ Center

**Figure 6:**
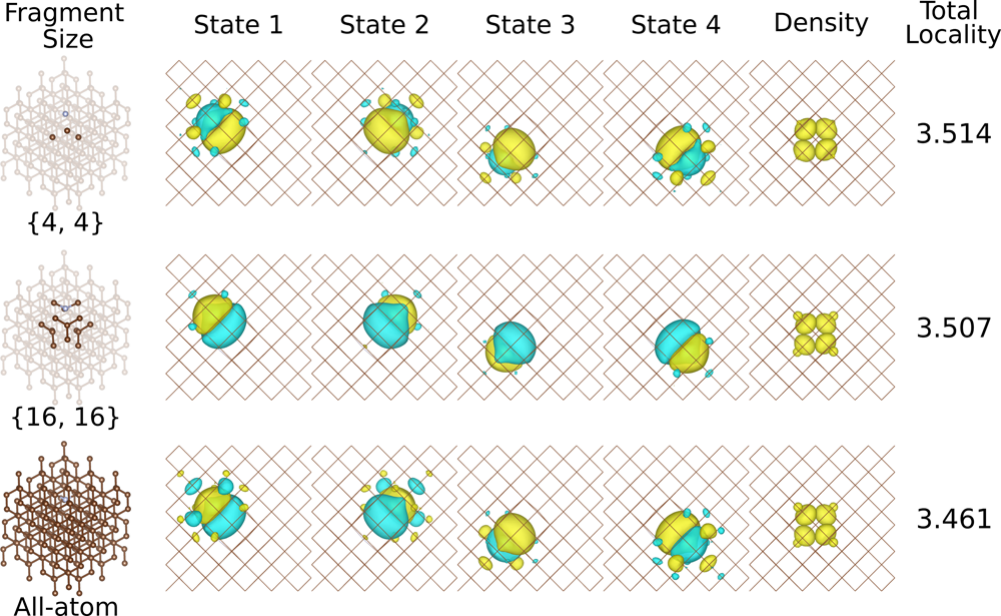
“p”-like Wannier function basis used in the Hubbard model calculations. Each row provides the fragment model, the corresponding set of PMWFs obtained from this fragment, the electron density constructed from these four PMWFs, and the total locality computed from eq 21. Here, the {4,4} fragment represents the minimal model, and the {16,16} one is found with the optimal fragment size. The isosurface value is set at 0.02.

To further demonstrate the practical application and quality of the sF-PMWF approach, we investigate the optical transitions in the NV^–^ center using the “p”-like Wannier functions (see Figure 6) that form a minimal basis. To model the excited states of the NV^–^ center, we solve the Hubbard Hamiltonian written as
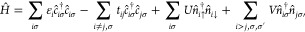
20where  and  are creation and annihilation operators in site *i* with spin σ, and  is a particle number operator. ε_*i*_ and *t*_*ij*_ are the on-site and hopping energies. *U* and *V* represent the on-site and intersite Coulomb interactions, respectively. It is a minimal model of the NV^–^ center that is commonly used^60−63^ to describe its low-lying excited states. Note, although including screening is important to capture the physics of the system correctly and has been extensively studied,^61,63,64^ only bare interactions are considered in this work to focus on the sensitivity to the variations of the Wannier basis. In this section, we will particularly comment on the selection of the fragment on which the electronic states are localized. Note that the fragment size is independent of the sF-PMWF methodology, but it represents an important parameter.

First, we focus on the results computed from the sF-PMWF Wannier basis of the four-atom fragment shown in Figure 6. The three lowest energy transitions are given in Table 3 in parentheses. For the 3D periodic systems, the two small cells slightly underestimate the ^3^*E* – ^3^*A*_2_ transition energy and overestimate the ^1^*A*_1_ – ^3^*A*_2_ one. Instead, the ^1^*E* – ^3^*A*_2_ transition converges well to the supercell size. The ^3^*E* – ^3^*A*_2_ and ^1^*A*_1_ – ^1^*E* transition energies are underestimated with respect to the experimental values of 1.95 and 1.19 eV, respectively. However, these results agree well with other theoretical calculations that employ PBE functionals to compute the bare Hubbard model parameters.^65−70^ The ^1^*E* – ^3^*A*_2_ transition energy fluctuates mildly with respect to the supercell size but maintains a comparable magnitude. The results computed in bulk systems from the sF-PMWF basis agree perfectly with the F-PMWF ones (see the F-PMWF results in Table S15), confirming the equivalency of the two sets of localized orbitals. In contrast, the F-PMWF and sF-PMWF differ slightly more from each other for the slab results. To investigate this difference in transition energies, we first examine the equivalence of the two sets of “p-like” Wannier functions: the orbitals from sF-PMWF have >99.99% overlap with their counterparts from F-PMWF, i.e., these two sets of states are practically identical. The numerical results are provided in Table S16. By subtracting corresponding sF-PMWF and F-PMWF Wannier orbitals, we observe seemingly negligible difference (slightly higher than the numerical noise), which however affects the Hubbard model calculations. Comparing the Hamiltonian computed with the two basis sets, we found that the discrepancy in transition energies stems only from the ionic part of the *t* parameters (see the definition of *t* parameters in eq S2 in the SI). In contrast, the kinetic part, sensitive to the small variation of the Wannier functions, is practically identical, confirming that both orbitals should be considered as equivalent. The small, ≪0.01%, difference is distributed over the real-space grid, and it becomes sizable enough for the slab calculation because of the system size (which is significantly larger than the bulk systems).

**Table 3:** Excited-State Transition Energies of the NV^–^ Center in the Four Investigated Systems Using the Wannier Function Basis Obtained from sF-PMWF Calculations^a^

	energy (eV)			
transition symmetry	215-atom cell	511-atom cell	999-atom cell	slab
^3^*E* – ^3^*A*_2_	2.108 (1.560)	2.277 (1.695)	2.312 (1.710)	1.343 (0.363)
^1^*A*_1_ – ^3^*A*_2_	1.433 (1.325)	1.310 (1.270)	1.202 (1.193)	1.159 (0.292)
^1^*E* – ^3^*A*_2_	0.447 (0.378)	0.435 (0.381)	0.413 (0.368)	0.329 (0.091)

a The numbers with and without the parentheses correspond to the {4,4} and {16,16} fragments, respectively.

Furthermore, the slab results are strikingly different from the bulk, i.e., the transition energies are up to 70%–80% lower than those in bulk. As we show below, this is due to the selection of the fragment size and independent of the completeness of the orbital space. To the best of our knowledge, we note that no calculations for shallow NV^–^ centers in slabs have been done previously. Hence, it is not possible to compare our results with any reference.

The situation is remedied when the fragment size effects are considered. As noted earlier, the fragment studied in the previous sections is actually a minimal model, i.e., the orbital localization is considered only on the four atoms where the “p”-like states are located, and the total number of orbitals on these four atoms is 16. However, neglecting the neighboring atoms might lead to a mixed character of “p”-like states and C–C (or N–C) covalent bonds. To test this, we investigate four combinations of  fragments: for instance, {4,16} represents the case where four atoms are considered in the folding step, while 16 atoms (including the bonded atoms) are considered in the unfolding step. A detailed investigation of the various parameters is performed on the 215-atom system. The corresponding fragments are presented in Figure S2. The four Wannier functions used for the Hubbard model are illustrated in Figure 6, where we compare the {4,4} fragment, the {16,16} fragment, and the all-atom case.

For a better comparison among different sets of PMWFs (Figure S18), we also provide the spatial overlaps between the fragmentation approaches and the all-atom calculation, |⟨ψ_*i*_|ψ_*j*_⟩|, in Table S13. The all-atom calculation refers to orbital localization on all atoms at once using G-PMWF. Numerically, the {4,16} combination gives the closest solutions to the all-atom ones. Note that in the all-atom case, the optimization does not preferentially localize single-electron states near the defect; rather, it seeks globally most localized states. Such an approach is not guaranteed to generate transformed PMWFs that are optimal for the mapping onto the Hubbard model. Indeed, we discuss this point in detail below.

In contrast, the results for the {4,4} combination represent the minimal fragment where the optimization is performed for 16 orbitals on four atoms neighboring the defect center. These PMWFs from the minimal model are shown in Figure 6 and display overlocalization of the “p”-like states in the NV^–^ center, i.e., the orbitals are less centered on the atoms and tend to merge at the geometric center. This is a purely numerical artifact of a too-small optimization space which is alleviated (Figure S18) when the 12 bonded atoms are included to compete with the geometric center for the electron density. Due to this, we disregard the {4,4} case further.

Upon visual inspection, the {16,16} combination *graphically* gives the most localized “p”-like orbitals (the second row in Figure 6). To provide a quantitative measure of localization, we calculate the locality of each “p”-like state on the corresponding atom plus its neighboring bonded atoms to account for the environment
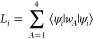
21where *i* denotes the *i*^th^ “p”-like state, and *A* sums over the four atoms (1 center atom + 3 bonded atoms). The value for each individual state is summarized in Table S14, where we use the sum, , to represent the whole set of PMWFs. In agreement with the visual analysis (Figure 6), the {16,16} combination exhibits the strongest localization attributed to the modification of the objective functional (eq 7). As commented on by Jónsson^22^ et al., the solutions to “maximally-localized Wannier functions” are actually not unique and sometimes ambiguous since the resulting localized orbitals are determined by the objective functional. We emphasize that the traditional G-PMWF approach evaluates the overall orbital localization on all the atoms, but it does not necessarily reach maximal localization on a specific subsystem (fragment). Instead, the proposed fragmentation treatment in this work leads to an objective functional for regionally localized orbitals. We surmise that this approach is more beneficial for effective embedding and downfolding.

To further analyze the results, we use the four sets of PMWFs and compute the optical transition energies for the 215-atom system (Table 4). We see that the ^3^*E* – ^3^*A*_2_ is the most sensitive to the basis, while the other two are less. The {4,16} combination provides results that are closest to the all-atom calculations. Compared with the most localized case ({16,16}), the other results are consistently underestimated by up to 0.55 eV. From these results, it is clear that the extent of orbital localization affects various observables differently. While some optical transitions for a given system are insensitive, others can be highly dependent on the basis. The sensible strategy is to search for a fragment that provides the maximal localization on each atom of interest and seek convergence of the observables of interest.

**Table 4:** Excited-State Transition Energies of the NV^–^ Center in the 215-Atom System Using the Wannier Function Basis Obtained from Different Sizes of the Fragment as well as the All-Atom Calculation

	energy (eV)				
transition symmetry	{4,4}	{4,16}	{16,16}	{40,40}	all-atom
^3^*E* – ^3^*A*_2_	1.560	1.770	2.108	1.860	1.715
^1^*A*_1_ – ^3^*A*_2_	1.325	1.373	1.433	1.384	1.355
^1^*E* – ^3^*A*_2_	0.378	0.407	0.447	0.417	0.398
	3.514	3.464	3.507	3.411	3.461

In the rest of the paper, we employ the {16,16} fragment to obtain the PMWF basis. The parameter study of orbital localization using this fragment is provided in Tables S17–S20.The excited-state transition energies are summarized in Table 3. For the bulk systems with the new “p”-like basis, the ^3^*E* – ^3^*A*_2_ transition gap is enlarged by up to 0.6 eV from the less localized basis, while the other two transition energies are relatively less sensitive to the change of basis.

The effect of the fragment size is most pronounced for the slab. If the {16,16} fragment is used, the results are similar to those for the bulk systems. In detail: the ^3^*E* – ^3^*A*_2_ transition is predicted ∼1 eV lower than that in bulk, while the other two are only slightly lower (by ∼0.1 eV) compared to the 999-atom cell. Here, the significant lowering of the triplet–triplet transition energy in the slab can be attributed to the interplay with the surface states of nitrogen-atom passivation layer. The surface states dive below the conduction band minimum of the bulk states, are located inside the band gap, and affect the position of the in-gap defect states. Finally, we remark that these observations underline the importance of fragment selection. However, they are completely independent of the proposed sequential exhausting methodology. Indeed, the results obtained with the sF-PMWF and F-PMWF methods agree excellently (Table S15) in each case, while the results depend on the fragment size.

## Conclusions and Perspective

By introducing the fragmentation treatment and the sequential exhaustion of the orbital space to the traditional F-PMWF method, we develop a swift, efficient, and robust algorithm, sF-PMWF, to obtain a set of regionally localized states on a subsystem of interest. The completeness and efficiency are insensitive to the choice of input parameters. The core idea is to reduce the dimensionality of matrices during the maximization process. The resulting scaling is reduced from being hyperquadratic to linear. For the applications of localized basis to the Hubbard model, the excited-state calculations are sensitive to the localized basis. While the Pipek-Mezey scheme is an ideal candidate to provide localized states with optimal localization for the whole system, it does not necessarily lead to “maximally” localized orbitals on a specific subsystem, but in our fragmentation treatment, one can carefully select the atoms (the strategy is mentioned above) to reach “maximally” localized orbitals on the subsystem and avoid the overlocalization issue.

The resulting sF-PMWF method has five primary benefits: (1) largely shortens the time per SA iteration and makes it easier to monitor the progress of localization; (2) significantly lowers the total job time and scaling for systems with thousands of electrons; (3) provides regionally localized orbitals with higher extent of localization; (4) is less demanding for computing resources, e.g., memory and CPUs; and (5) can be performed without the knowledge of canonical eigenstates if it is coupled with stochastic methods (e.g., stochastic DFT). The stochastic basis search approach exhibits higher efficiency than the traditional method for systems with over 4000 electrons.

Furthermore, we want to comment on the following prospective applications of the sequential exhausting method: (1) This method can be generalized to obtain localized states of the whole system. Given that the rest space can always be updated or reconstructed by Gram-Schmidt orthogonalization, the sF-PMWF calculation can then be sequentially applied to all the fragments in the entire system. (2) This method can be coupled with other maximizers, e.g., conjugated gradient and BFGS approach, to further facilitate the convergence of the PM functional. (3) The idea of sequentially exhausting the orbital space can be also implemented in other localization schemes, e.g., Foster-Boys, for a suitably defined fragment and an associated cost function.

We believe that the sF-PMWF method will find numerous applications in condensed matter problems, either in chemistry, materials science, or computational materials physics.

## Supporting Information Available

Texts: downfolded effective Hamiltonian for Hubbard model, excited states of NV^–^ center, preparation of stochastic basis using deterministic eigenstates; Figures: three fragments of different sizes and all-atom system exemplied by 215-atom cell, scaling of *t*^*outer*^ with respect to *N*_*w*_, evolution of objective functional with respect to outer-loop step using deterministic and stochastic basis, investigations of (*N*_*c*_, *N*_*r*_) on 511-atom system, total wall time of orbital localization on four investigated systems, relative number of SA iterations in sF-PMWF calculations for 215- and 511-atom systems, scaling of time per iteration with respect to *N*_*s*_, electron density localized on NV^–^ center of three solid systems, four “p-like” localized Wannier functions on NV^–^ center of three solid systems, electron density localized on arbitrary carbon atom of four investigated systems, four “p-like” localized Wannier functions as well as resulting electron density on NV^–^ center of 215-atom system using different sizes of fragments; Tables: transition energies obtained from different Wannier functions basis using energy-windowing truncated space, comparison of F-PMWF and sF-PMWF calculations with different *N*_*c*_ and *N*_*r*_ for four investigated systems using 4-atom fragment, comparison of time spent on folding and unfolding steps, time per outer-loop iteration and normalized time per outer-loop iteration in sF-PMWF calculations of four investigated systems, time per macro-cycle iteration and normalized time per macro-cycle iteration in sF-PMWF calculations of four investigated systems, total wall time and normalized total wall time of F-PMWF and sF-PMWF calculations for four investigated systems, time per steep-ascent step in F-PMWF and sF-PMWF calculations, number of iterations required to reach convergence for four investigated systems, converged maximized PM functional values, spatial overlap between Wannier functions obtained from F-PMWF and sF-PMWF calculations for 2303-atom slab, spatial overlap between Wannier functions obtained from fragment approaches with those obtained from all-atom calculation, transition energies of four investigated systems using Wannier functions basis obtained from F-PMWF calculations, comparison of F-PMWF and sF-PMWF calculations with different *N*_*c*_ and *N*_*r*_ for four investigated systems using 16-atom fragment (PDF)
