# Supplementary material for: Reduced Scaling of Optimal Regional Orbital Localization via Sequential Exhaustion of the Single-Particle Space
Source: J Chem Theory Comput. 2022 Jul 11;18(8):4960–72. doi: 10.1021/acs.jctc.2c00315 (PMC9367006; doi:10.1021/acs.jctc.2c00315)
Supplement: Supplementary file 1 — ct2c00315_si_001.pdf [file ct2c00315_si_001.pdf]

Supporting information for “Reduced scaling of optimal regional orbital localization via sequential exhaustion of the single-particle space”

Guorong Weng, Mariya Romanova, Arsineh Apelian, Hanbin Song, and Vojtěch Vlček*

Department of Chemistry and Biochemistry, University of California, Santa Barbara, CA 93106-9510, U.S.A.

E-mail: vlcek@ucsb.edu

Downfolded effective Hamiltonian

In large systems with a certain anisotropy (defects in semiconductors, molecules in solvent environments) all physical phenomena can be attributed to a small active space embedded in a host environment. Thus, it is common to map the problem onto the effective Hamiltonian, defined within an active space.

$$\begin{aligned}\hat{H} = \sum_{i\sigma} \varepsilon_i \hat{c}^\dagger_{i\sigma} \hat{c}_{i\sigma} - \sum_{i\neq j,\sigma} t_{ij} \hat{c}^\dagger_{i\sigma} \hat{c}_{j\sigma} + \\ + \sum_{i\sigma} U \hat{n}^\dagger_{i\uparrow} \hat{n}_{i\downarrow} + \sum_{i>j,\sigma,\sigma'} V \hat{n}^\dagger_{i\sigma} \hat{n}_{j\sigma'},\end{aligned} \tag{S1}$$

where $\hat{c}^\dagger_{i\sigma}$ and $\hat{c}_{i,\sigma}$ are creation and annihilation operators in site i with spin σ and $\hat{n}^\dagger_{i\sigma}$ is a particle number operator. The ε_i, t_{ij} are the on-site and hopping energies.

We extract the Hamiltonian parameters ε, t U_i and V_{ij} from the first-principles calcula-

tions employing large supercells. To compute the onsite and hopping parameters we calculate the integral containing kinetic and ionic potential terms:

$$
\begin{aligned}
\varepsilon_i &= \int \varphi_i^*(\mathbf{r})[-\frac{1}{2}\nabla^2 + V^{\text{ion}}]\varphi_i(\mathbf{r})d\mathbf{r} \\
t_{ij,i\neq j} &= \int \varphi_i^*(\mathbf{r})[-\frac{1}{2}\nabla^2 + V^{\text{ion}}]\varphi_j(\mathbf{r})d\mathbf{r}
\end{aligned}
\tag{S2}
$$

The U_i represents Coulomb on-site interactions of electrons with a different spin, while V_{ij} is the Coulomb inter-site interaction, which we compute as:

$$
\begin{aligned}
U_i &= \int \varphi_i^*(\mathbf{r})\varphi_i(\mathbf{r})V(\mathbf{r},\mathbf{r}')\varphi_i^*(\mathbf{r}')\varphi_i(\mathbf{r}')d\mathbf{r}d\mathbf{r}' \\
V_{ij} &= \int \varphi_i^*(\mathbf{r})\varphi_i(\mathbf{r})V(\mathbf{r},\mathbf{r}')\varphi_j^*(\mathbf{r}')\varphi_j(\mathbf{r}')d\mathbf{r}d\mathbf{r}'
\end{aligned}
\tag{S3}
$$

where, the $V(\mathbf{r},\mathbf{r}')$ is the bare Coulomb interaction.

Excited states of the NV$^-$ center

Table S1 shows the excited states of the NV$^-$ center computed in the basis of the Wannier functions that were obtained with different energy windows. The full space energy window is $\sim$ 24 eV below the Fermi energy. One can see that even 20 eV window results in an extremely underestimated result, while for 10 eV window the order of states is reversed. As a measure of the localization we report the value of the objective functional $\mathcal{P}'$ (see main text). The $\mathcal{P}'$ is set to 100% for case where the full space is used in the energy window.

Table S1: Comparison of the excited-state transition energies of the NV^- center in the 511-atom system with various truncated orbital space for the localization.

Symmetry	10 eV	20 eV	full space
$^3E - {^3A_2}$	0.121	1.003	1.556
$^1A_1 - {^3A_2}$	0.156	0.947	1.324
$^1E - {^3A_2}$	0.039	0.259	0.378
$\mathcal{P}'$ (%)	49.5	86.6	100

Preparation of stochastic basis using deterministic eigenstates

The stochastic basis representing the complement (rest) space in our sF-PMWF calculations is prepared in a three-step manner. First, a random vector is constructed in the full space

$$|\zeta_i^m\rangle = \sum_{j=1}^{N_s} \alpha_{ij}^m |\phi_j\rangle , \tag{S4}$$

where m denotes the mth iteration in the outer-loop and $|\phi_j\rangle$ is the eigenstate in the full space. The set of random coefficients $\{\alpha_{ij}^m\}$ are associated with the outer-loop step m, i.e., a different m corresponds to a different set of coefficients.

The second step is to perform Gram-Schmidt orthogonalization such that the stochastic basis is orthogonal to the core space

$$|\zeta_i^m\rangle = |\zeta_i^m\rangle - \sum_{k=1}^{N_c} \frac{\langle\psi_k^c|\zeta_i^m\rangle}{\langle\psi_k^c|\psi_k^c\rangle} |\psi_k^c\rangle , \tag{S5}$$

where $|\psi_k^c\rangle$ represents the state in the core space. The stochastic basis is then made mutually orthogonal

$$|\zeta_i^m\rangle = |\zeta_i^m\rangle - \sum_{j=1}^{i-1} \frac{\langle\zeta_j^m|\zeta_i^m\rangle}{\langle\zeta_j^m|\zeta_j^m\rangle} |\zeta_j^m\rangle \qquad i \geq 2. \tag{S6}$$

The last step is to normalize the stochastic basis such that

$$\langle\zeta_i^m|\zeta_j^m\rangle = \delta_{ij} \tag{S7}$$

and

$$\langle \psi_i^c | \zeta_j^m \rangle = 0. \tag{S8}$$

After these three steps, the construction of stochastic basis for the mth step is completed and it is ready to enter the work space.

Supplementary Tables and Figures

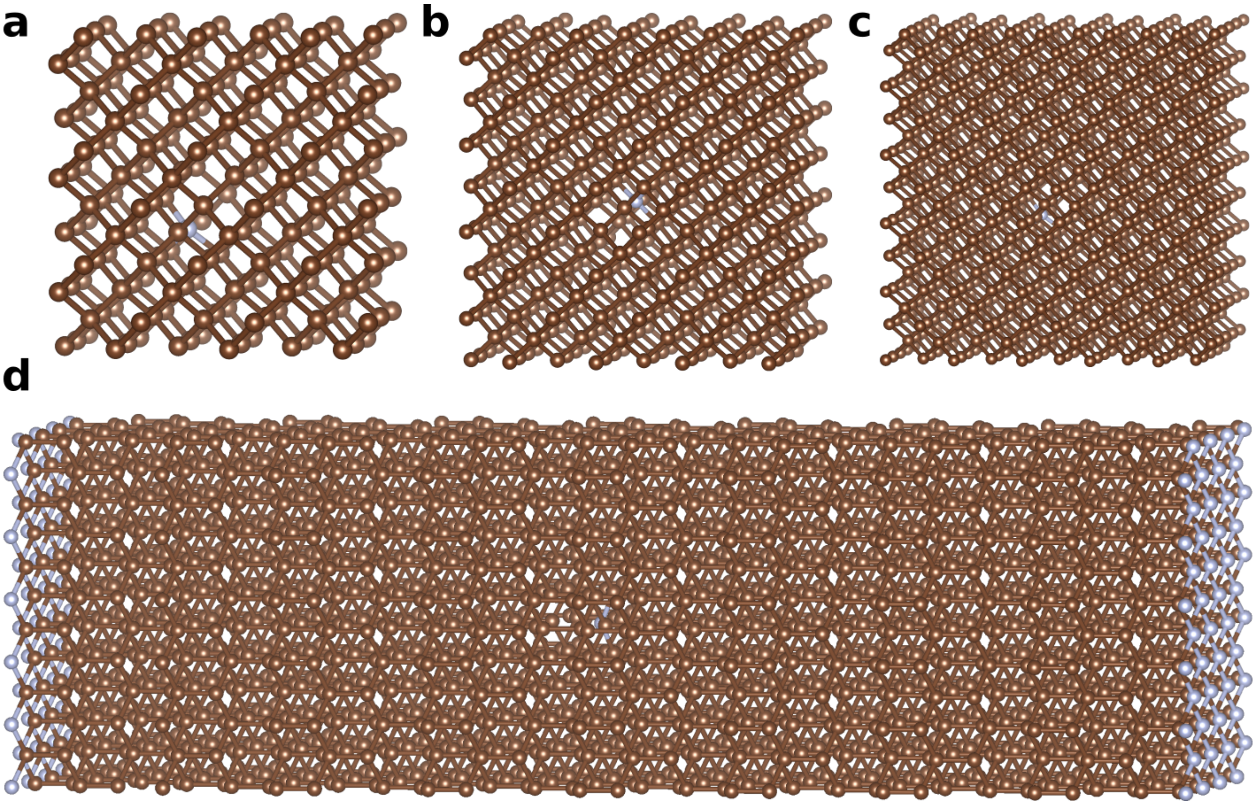

Figure S1: Chemical structures of the four investigated diamond with NV^- center systems: (a) 215-atom supercell; (b) 511-atom supercell; (c) 999-atom supercell; (d) 2303-atom slab.

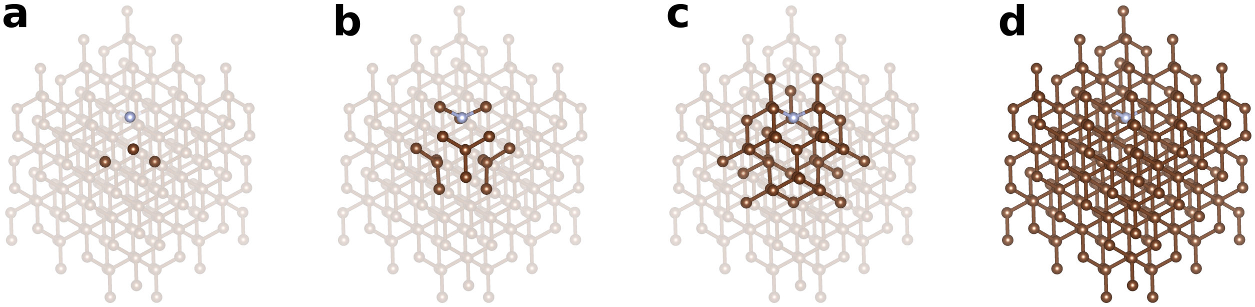

Figure S2: Composition of the three fragments as well as the all-atom system: (a) 4-atom fragment; (b) 16-atom fragment; (c) 40-atom fragment; (d) all-atom system. The fragments are exemplified using the 215-atom cell while each fragment is found extremely similar around the NV^- center in the other investigated systems.

Table S2: Comparison of sF-PMWF and F-PMWF with different combinations of N_c and N_r for orbital localization on the 215-atom system

Method	N_c	N_r	N_w	N_b	N_{it}^{outer}	Converged $\mathcal{P}'$	Converged $\mathcal{P}$	$\mathcal{P}'$ after 1st cycle (percentage gained)	t^{outer} (s)	t^{macro} (s)	n^{macro}	Total wall time (s)
F-PMWF	-	-	-	-	1	4.9345	4.6656	-	-	-	-	308
sF-PMWF	16	4	20	104	520	4.9436	4.6656	4.7882 (97%)	0.09	9.07	5	47
sF-PMWF	16	8	24	42	260	4.9436	4.6656	4.8366 (98%)	0.11	5.87	5	31
sF-PMWF	16	16	32	26	156	4.9435	4.6655	4.6533 (94%)	0.17	4.65	6	29
sF-PMWF	16	32	48	13	65	4.9436	4.6656	4.7039 (95%)	0.32	4.19	5	22
sF-PMWF	16	48	64	9	54	4.9436	4.6656	4.6888 (95%)	0.50	4.51	6	28
sF-PMWF	16	64	80	7	35	4.9436	4.6656	4.7348 (96%)	0.73	5.09	5	27
sF-PMWF	24	24	48	17	68	4.9346	4.6656	4.7523 (96%)	0.37	6.31	4	26
sF-PMWF	32	16	48	25	100	4.9346	4.6657	4.8425 (98%)	0.29	7.21	4	30
sF-PMWF	40	8	48	49	245	4.9346	4.6657	4.8868 (99%)	0.29	14.06	5	72
sF-PMWF (stochastic)	16	32	48	-	216	4.9346	4.6656	-	3.47	-	-	729

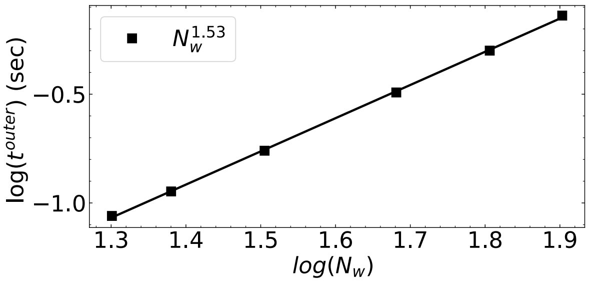

Figure S3: The log of the time per outer-loop iteration (t^{outer}) as a function of the log of the number of states in the work space (N_w). The scaling of t^{outer} with N_w is derived from the slope of the linear fitting.

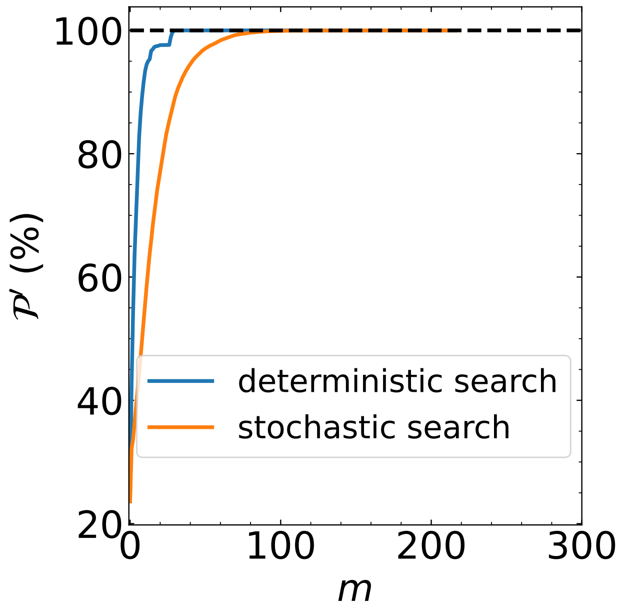

Figure S4: Convergence of the functional $\mathcal{P}'$ with respect to the outer-loop step m for the NV^- center of the 215-atom system. Blue curve: localization performed with deterministic basis in the rest space. Orange curve: localization performed with stochastic basis in the rest space. The (16,32) combination is employed in both calculations.

Table S3: Comparison of sF-PMWF and F-PMWF with different combinations of N_c and N_r for orbital localization on the 511-atom system

Method	N_c	N_r	N_w	N_b	N_{it}^{outer}	Converged $\mathcal{P}'$	Converged $\mathcal{P}$	$\mathcal{P}'$ after 1st cycle (percentage gained)	t^{outer} (s)	t^{macro} (s)	n^{macro}	Total wall time (s)
F-PMWF	-	-	-	-	1	4.9222	4.6498	-	-	-	-	7360
sF-PMWF	16	4	20	252	1512	4.9223	4.6498	4.6254 (94%)	0.26	64.97	6	397
sF-PMWF	16	8	24	126	630	4.9223	4.6498	4.6392 (94%)	0.35	44.65	5	230
sF-PMWF	16	16	32	63	315	4.9223	4.6498	4.6074 (94%)	0.53	34.44	5	175
sF-PMWF	16	32	48	21	128	4.9223	4.6498	4.634 (94%)	0.83	26.67	4	114
sF-PMWF	16	48	64	21	126	4.9223	4.6498	4.7189 (96%)	1.12	23.52	6	148
sF-PMWF	24	24	48	42	252	4.9223	4.6498	4.8208 (98%)	0.86	36.75	6	224
sF-PMWF	32	16	48	62	310	4.9223	4.6498	4.8476 (98%)	0.83	51.56	5	265
sF-PMWF	40	8	48	123	738	4.9223	4.6498	4.8791 (99%)	0.83	102.13	6	621

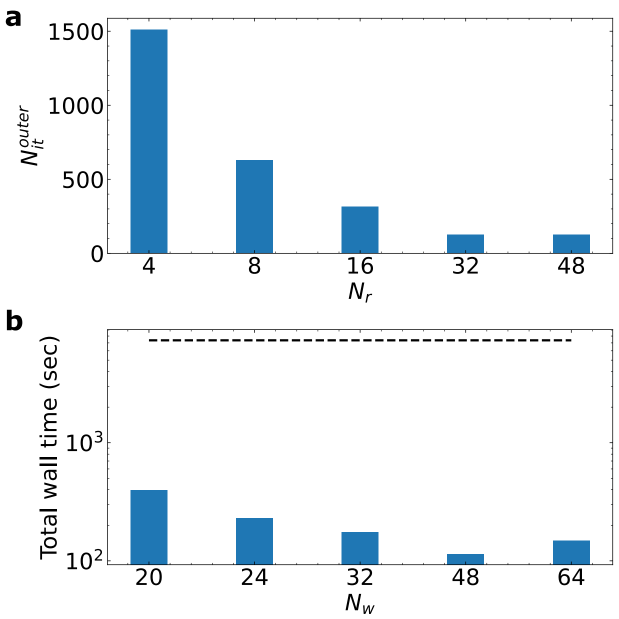

Figure S5: Investigation of different combinations of N_c and N_r for the localization on the NV^- center of the 511-atom cell. N_c is fixed at 16. (a) Total number of iteration steps in the outer-loop as a function of the N_r. (b) Total wall time of the calculation as a function of N_w. Dashed line indicates the total wall time from the F-PMWF method using the full orbital space.

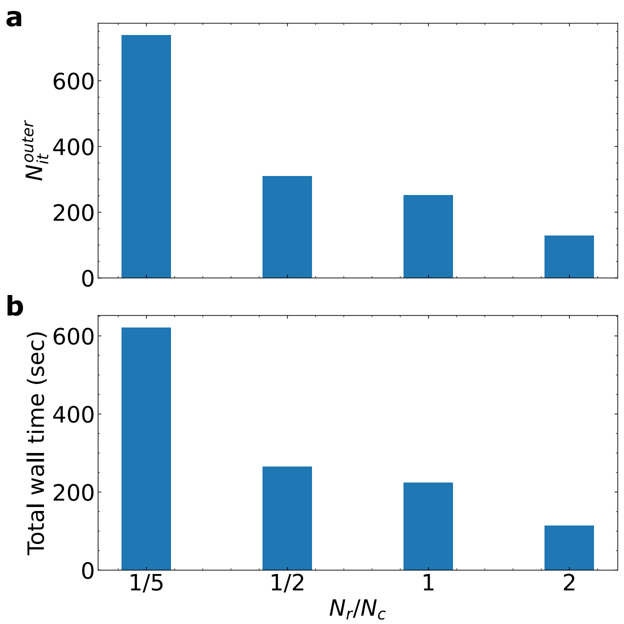

Figure S6: Investigation of different combinations of N_c and N_r for the localization on the NV^- center of the 511-atom cell. N_w is fixed at 48. (a) Total number of iteration steps in the outer-loop as a function of the N_r/N_c ratio; (b) The total wall time as a function of the N_r/N_c ratio.

Table S4: Comparison of sF-PMWF and F-PMWF with different combinations of N_c and N_r for orbital localization on the 999-atom system

Method	N_c	N_r	N_w	N_b	N_{it}^{outer}	Converged $\mathcal{P}'$	Converged $\mathcal{P}$	$\mathcal{P}'$ after 1st cycle (percentage gained)	t^{outer} (s)	t^{macro} (s)	n^{macro}	Total wall time (s)
F-PMWF	-	-	-	-	1	4.9194	4.6447	-	-	-	-	42006
sF-PMWF	16	16	32	124	620	4.9194	4.6446	4.6175 (94%)	0.89	109.89	5	575
sF-PMWF	16	32	48	62	310	4.9194	4.6446	4.6089 (94%)	1.50	92.81	5	489
sF-PMWF	16	48	64	42	210	4.9195	4.6446	4.0349 (82%)	2.22	93.47	5	493
sF-PMWF (stochastic)	16	32	48	-	999	4.9194	4.6446	-	23.78	-	-	24172

Table S5: Comparison of sF-PMWF and F-PMWF with different combinations of N_c and N_r for orbital localization on the 2303-atom slab system

Method	N_c	N_r	N_w	N_b	N_{it}^{outer}	Converged $\mathcal{P}'$	Converged $\mathcal{P}$	$\mathcal{P}'$ after 1st cycle (percentage gained)	t^{outer} (s)	t^{macro} (s)	n^{macro}	Total wall time (s)
F-PMWF	-	-	-	-	1	4.9414	4.6731	-	-	-	-	695370
sF-PMWF	16	16	32	290	1740	4.9414	4.6731	3.7145 (75%)	1.11	333.03	6	2644
sF-PMWF	16	32	48	145	870	4.9414	4.6731	3.6976 (75%)	1.84	266.02	6	1683
sF-PMWF	16	48	64	97	582	4.9414	4.6731	3.8859 (79%)	2.50	241.98	6	1538

Table S6: Time spent on the folding and unfolding steps of the four investigated systems. The unfolding step of each calculation employs the (16,32) combination.

System	Time (s)	
	Folding step	Unfolding step
215-atom cell	22	0.52
511-atom cell	114	1.85
999-atom cell	489	6.90
slab	1683	17.79

Table S7: Information of the four investigated systems as well as the time and normalized time per outer-loop iteration and per macro-cycle.

System	N_e	N_s	N_g	t^{outer} (s)	t_n^{outer} (s)	t^{macro} (s)	t_n^{macro} (s)	n^{macro}
215-atom cell	864	432	314432	0.32	1.99	4.19	25.83	5
511-atom cell	2048	1024	778688	0.83	2.08	26.67	66.45	4
999-atom cell	4000	2000	1404928	1.50	2.07	92.81	128.16	5
slab	9312	4656	1940120	1.89	1.89	266.02	266.02	6

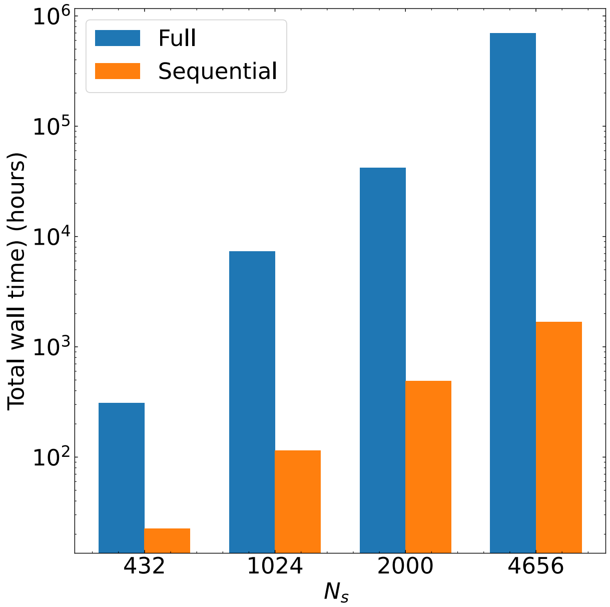

Figure S7: Total wall time of orbital localization on each system with respect to the number of occupied states N_s. Blue bar: F-PMWF using the full orbital space. Orange Bar: sF-PMWF using the work space.

Table S8: Total wall time and normalized total wall time of four investigated systems.

System	Total wall time (s)		Normalized total wall time (s)	
	F-PMWF	sF-PMWF	F-PMWF	sF-PMWF
215-atom cell	308	22	1903	139
511-atom cell	7360	114	18339	284
999-atom cell	42006	489	58007	675
slab	695370	1683	695370	1683

Table S9: Time per SA iteration step in F-PMWF and sF-PMWF calculations for the four investigated systems

System	Time per SA iteration (s)	
	F-PMWF	sF-PMWF
215-atom cell	0.29	5.28×10^{-4}
511-atom cell	8.62	5.11×10^{-4}
999-atom cell	61.80	5.13×10^{-4}
slab	1056.26	4.94×10^{-4}

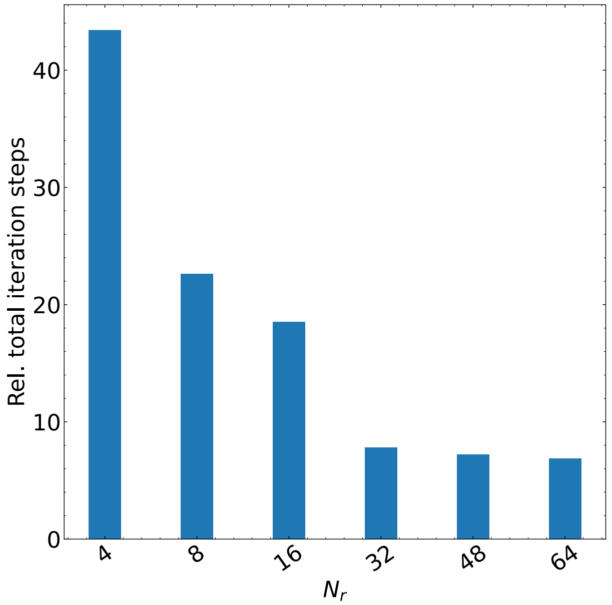

Figure S8: Number of total SA iteration steps in sF-PMWF calculation relative to the number of total SA iteration steps in the F-PMWF calcuation for the 215-atom system using different N_r. The N_c is fixed at 16.

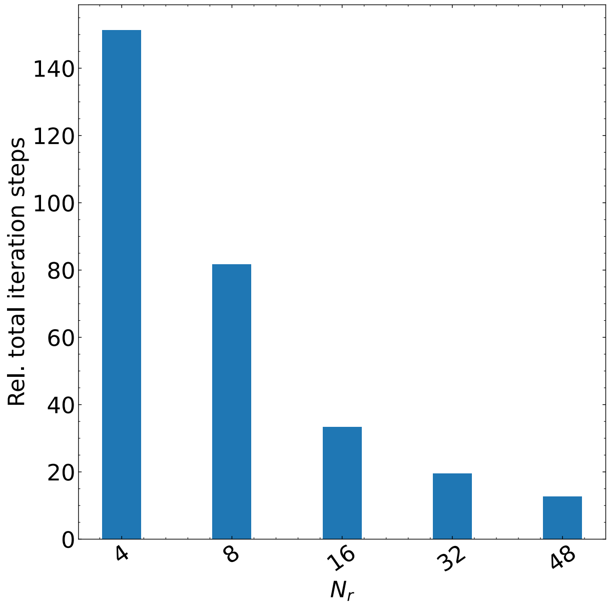

Figure S9: Number of total SA iteration steps in sF-PMWF calculation relative to the number of total SA iteration steps in the F-PMWF calculation for the 511-atom system using different N_r. The N_c is fixed at 16.

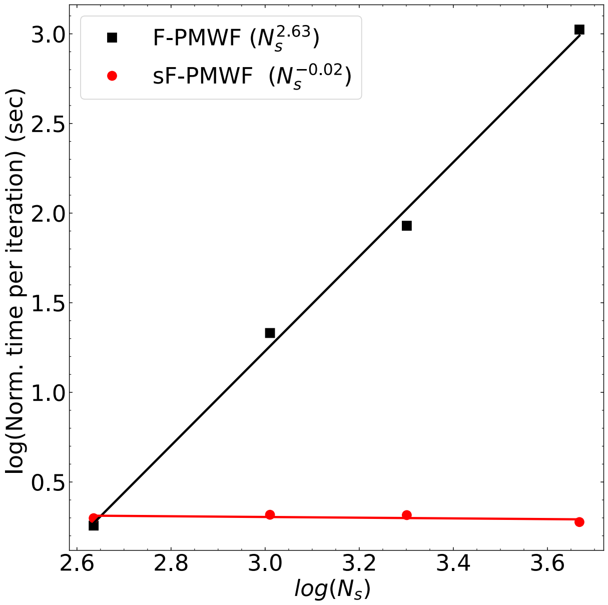

Figure S10: The log of the normalized time per iteration plotted as a function of the log of number of occupied states N_s for the four investigated systems. The black line and square points represent the normalized t^{SA} obtained from the F-PMWF method using the full orbital space. The red line and circle points represent the normalized t^{outer} obtained from the sF-PMWF method using the constructed work space. The time per iteration is normalized to the largest grid (2303-atom system). The scaling is derived from the slope of each fitting.

Table S10: Number of iterations required to reach convergence in F-PMWF and sF-PMWF calculations.

System	N_{it}^{SA} in F-PMWF	N_{it}^{outer} in sF-PMWF
215-atom cell	637	65
511-atom cell	700	128
999-atom cell	586	310
slab	650	870

Table S11: Converged maximized $\mathcal{P}'$ from F-PMWF and sF-PMWF calculations. The (16,32) combination is used in the sF-PMWF calculations.

system	Converged $\mathcal{P}'$	
	F-PMWF	sF-PMWF
215-atom cell	4.9345	4.9346
511-atom cell	4.9222	4.9223
999-atom cell	4.9194	4.9195
slab	4.9414	4.9414

Table S12: Converged maximized $\mathcal{P}$ from F-PMWF and sF-PMWF calculations.

system	Converged $\mathcal{P}$	
	F-PMWF	sF-PMWF
215-atom cell	4.6656	4.6656
511-atom cell	4.6498	4.6498
999-atom cell	4.6447	4.6446
slab	4.6731	4.6731

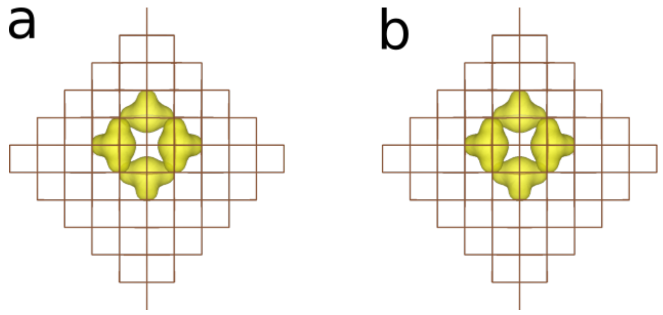

Figure S11: Electron density constructed from the 16 regionally localized states on the NV^- center of the 215-atom system: (a) F-PMWF; (b) sF-PMWF. The isosurface value is set at 0.05 for all the plots.

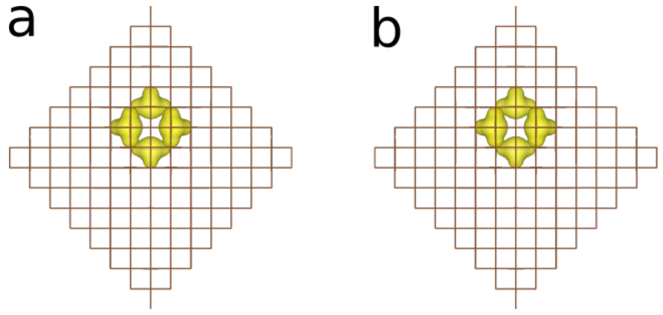

Figure S12: Electron density constructed from the 16 regionally localized states on the NV^- center of the 511-atom system: (a) F-PMWF; (b) sF-PMWF. The isosurface value is set 0.05 for all the plots.

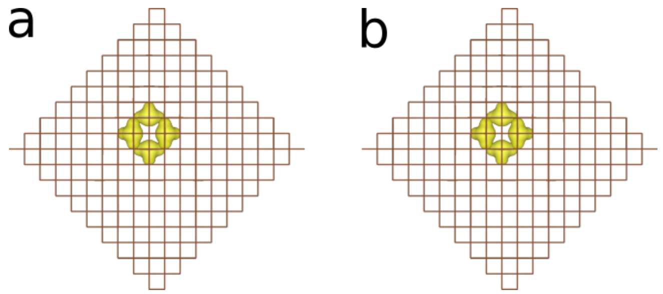

Figure S13: Electron density constructed from the 16 regionally localized states on the NV^- center of the 999-atom system: (a) F-PMWF; (b) sF-PMWF. The isosurface value is set at 0.05 for all the plots.

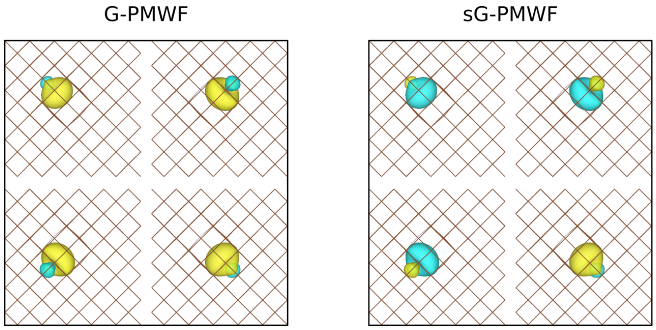

Figure S14: The 4 regionally localized "p"-like states around the NV^- center of the 215-atom system. The left 4 states are obtained from F-PMWF and the right 4 are obtained from sF-PMWF. The yellow and blue colors represent the phases of the single-particle wavefunction. The isosurface value is set 0.05 for all the plots.

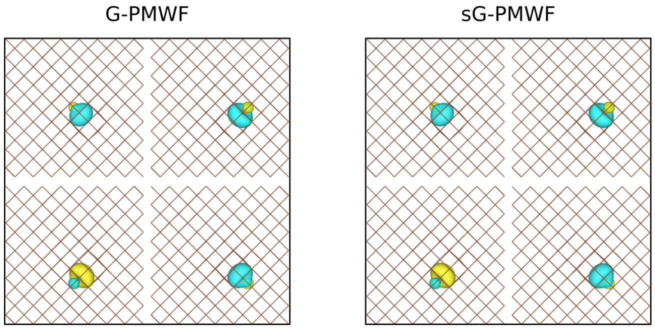

Figure S15: The 4 regionally localized "p"-like states around the NV^- center of the 511-atom system. The left 4 states are obtained from F-PMWF and the right 4 are obtained from sF-PMWF. The yellow and blue colors represent the phases of the single-particle wavefunction. The isosurface value is set at 0.05 for all the plots.

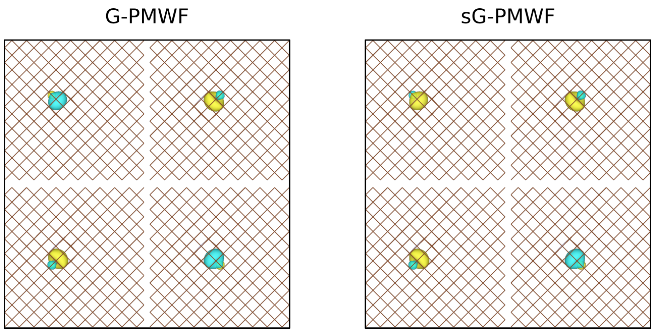

Figure S16: The 4 regionally localized "p"-like states around the NV^- center of the 999-atom system. The left 4 states are obtained from F-PMWF and the right 4 are obtained from sF-PMWF. The yellow and blue colors represent the phases of the single-particle wavefunction. The isosurface value is set at 0.05 for all the plots.

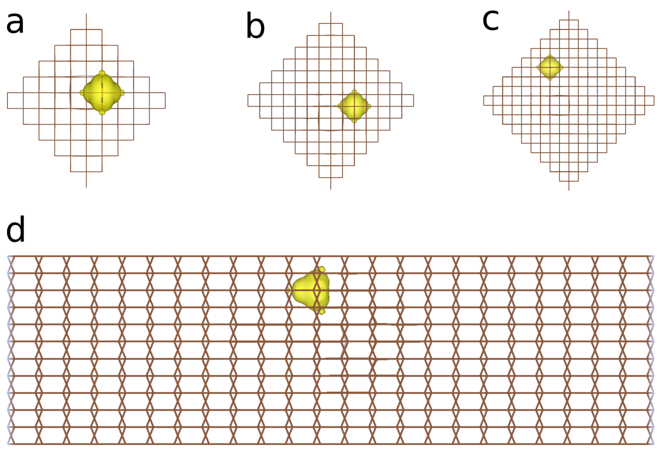

Figure S17: Electron density constructed from the 4 regionally localized states on an arbitrary carbon of the four investigated systems: (a) 215-atom system; (b) 511-atom system; (c) 999-atom system; (d) 2303-atom system. The isosurface value is set at 0.01 for all the plots.

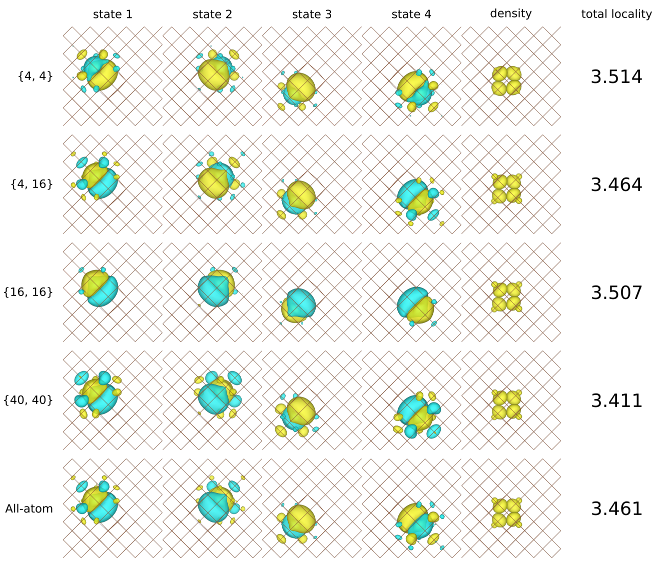

Figure S18: The 4 regionally localized "p"-like states around the NV^- center of the 215-atom system using different sizes of the fragment or using all the atoms. The last column shows the electron density constructed from these 4 states in each calculation. The isosurface value is set at 0.02 for all the plots.

Table S13: The spatial overlap between the set of Wannier basis from the fragment approaches and the set from the all-atom calculation.

Entry	state 1	state 2	state 3	state 4
{4,4}	0.981877	0.978799	0.978799	0.978799
{4,16}	0.999874	0.999704	0.999704	0.999704
{16,16}	0.991859	0.985258	0.985259	0.985259
{40,40}	0.997118	0.993832	0.993832	0.993789

Table S14: The locality of each set of Wannier function basis on the corresponding atom plus the neighoring bonded atoms.

Entry	state 1	state 2	state 3	state 4	$\sum L_i$
{4,4}	0.925726	0.862677	0.862680	0.862677	3.513760
{4,16}	0.915932	0.849211	0.849211	0.849209	3.463562
{16,16}	0.922626	0.861534	0.861534	0.861534	3.507228
{40,40}	0.908731	0.834126	0.833826	0.834018	3.410700
all-atom	0.915629	0.848489	0.848488	0.848488	3.461094

Table S15: Excited-state transition energies of the NV^- center in the four investigated systems using the Wannier function basis obtained from F-PMWF calculations. The numbers with and without the parenthesis correspond to the {4,4} and {16,16} fragment, respectively.

Transition	Energy (eV)			
symmetry	215-atom cell	511-atom cell	999-atom cell	slab
^3E – $^3A_2$	2.108 (1.560)	2.279 (1.695)	2.312 (1.710)	1.343 (0.399)
$^1A_1$ – $^3A_2$	1.433 (1.325)	1.310 (1.270)	1.202 (1.193)	1.159 (0.324)
^1E – $^3A_2$	0.447 (0.378)	0.435 (0.381)	0.413 (0.368)	0.329 (0.101)

Table S16: The spatial overlap between the two sets of "p-like" Wannier functions obtained from the sF-PMWF (ψ^s) method and the F-PMWF (ψ) method for the 2303-atom system.

	ψ_1^s	ψ_2^s	ψ_3^s	ψ_4^s
ψ_1	0.9999798	1.11×10^{-3}	2.68×10^{-4}	7.89×10^{-4}
ψ_2	1.11×10^{-3}	0.9999992	9.19×10^{-6}	1.33×10^{-6}
ψ_3	2.64×10^{-4}	9.33×10^{-6}	0.9999997	7.12×10^{-6}
ψ_4	7.93×10^{-4}	2.65×10^{-6}	6.89×10^{-6}	0.9999995

Table S17: Comparison of sF-PMWF and F-PMWF with different combinations of N_c and N_r for orbital localization on the 215-atom system with 16 atoms in the fragment

Method	N_c	N_r	N_w	N_b	N_{it}^{outer}	Converged $\mathcal{P}'$	Converged $\mathcal{P}$	$\mathcal{P}'$ after 1st access (percentage gained)	t^{outer} (s)	Total wall time (s)
F-PMWF	-	-	-	-	1	13.9402	6.7472	-	-	356
sF-PMWF	16	4	20	104	1664	13.9403	6.7472	12.3286 (88%)	0.10	170
sF-PMWF	16	8	24	42	676	13.9398	6.7471	12.3630 (87%)	0.14	96
sF-PMWF	16	16	32	26	182	13.9381	6.7466	11.9140 (85%)	0.23	43
sF-PMWF	16	32	48	13	104	13.9361	6.7463	11.9952 (86%)	0.43	46
sF-PMWF	16	48	64	9	81	13.9398	6.7471	12.5275 (90%)	0.58	48
sF-PMWF	16	64	80	7	42	13.9400	6.7471	12.9316 (93%)	1.02	44
sF-PMWF	16	80	96	6	36	13.9402	6.7471	13.5550 (97%)	1.36	51
sF-PMWF	32	48	80	9	90	13.9403	6.7472	13.6312 (98%)	0.72	66
sF-PMWF	48	32	80	12	108	13.9403	6.7472	13.9004 (99%)	0.75	82
sF-PMWF	64	16	80	23	184	13.9403	6.7472	13.9268 (99%)	0.71	132

Table S18: Comparison of sF-PMWF and F-PMWF with different combinations of N_c and N_r for orbital localization on the 511-atom system with 16 atoms in the fragment

Method	N_c	N_r	N_w	N_b	N_{it}^{outer}	Converged $\mathcal{P}'$	Converged $\mathcal{P}$	$\mathcal{P}'$ after 1st access (percentage gained)	t^{outer} (s)	Total wall time (s)
F-PMWF	-	-	-	-	1	13.9227	6.7261	-	-	10631
sF-PMWF	16	8	24	126	2142	13.9227	6.7260	11.2259 (81%)	0.34	732
sF-PMWF	16	16	32	63	882	13.9226	6.7261	11.1987 (80%)	0.46	416
sF-PMWF	16	32	48	32	352	13.9225	6.7260	11.3184 (81%)	0.74	269
sF-PMWF	16	48	64	21	210	13.9216	6.7258	12.3472 (89%)	1.21	263
sF-PMWF	16	64	80	16	160	13.9222	6.7260	11.5273 (83%)	1.57	259
sF-PMWF	16	80	96	13	104	13.9225	6.7260	12.0496 (86%)	2.13	230
sF-PMWF	16	96	112	11	77	13.9226	6.7261	12.6309 (91%)	3.00	240
sF-PMWF	16	112	128	9	90	13.9225	6.7260	13.2126 (95%)	4.06	374
sF-PMWF	32	48	80	21	252	13.9226	6.7260	13.6220 (98%)	1.54	398
sF-PMWF	48	32	80	31	186	13.9226	6.7260	13.8586 (99%)	1.47	283
sF-PMWF	64	16	80	60	300	13.9227	6.7260	13.8899 (99%)	1.42	434

Table S19: Comparison of sF-PMWF and F-PMWF with different combinations of N_c and N_r for orbital localization on the 999-atom system with 16 atoms in the fragment

Method	N_c	N_r	N_w	N_b	N_{it}^{outer}	Converged $\mathcal{P}'$	Converged $\mathcal{P}$	$\mathcal{P}'$ after 1st access (percentage gained)	t^{outer} (s)	Total wall time (s)
F-PMWF	-	-	-	-	1	13.9167	6.7200	-	-	58937
sF-PMWF	16	32	48	62	1178	13.9169	6.7198	11.0295 (79%)	1.53	1832
sF-PMWF	16	48	64	42	672	13.9172	6.7199	11.1849 (80%)	2.09	1435
sF-PMWF	16	64	80	31	310	13.9159	6.7195	11.3795 (82%)	3.06	978
sF-PMWF	16	80	96	25	200	13.9161	6.7195	11.6643 (84%)	4.06	840
sF-PMWF	16	96	112	21	189	13.9160	6.7195	12.1530(87%)	5.52	1074
sF-PMWF	16	128	144	16	128	1.9169	6.7198	12.2390 (88%)	8.74	1148

Table S20: Comparison of sF-PMWF and F-PMWF with different combinations of N_c and N_r for orbital localization on the 2303-atom system with 16 atoms in the fragment

Method	N_c	N_r	N_w	N_b	N_{it}^{outer}	Converged $\mathcal{P}'$	Converged $\mathcal{P}$	$\mathcal{P}'$ after 1st access (percentage gained)	t^{outer} (s)	Total wall time (s)
F-PMWF	-	-	-	-	1	13.9451	6.7539	-	-	761005
sF-PMWF	16	32	48	145	1595	13.9451	6.7539	12.2360 (88%)	1.75	2888
sF-PMWF	16	48	64	97	970	13.9451	6.7539	12.3156 (88%)	2.44	2454
sF-PMWF	16	64	80	73	730	13.9450	6.7540	12.2248 (88%)	3.66	2761
sF-PMWF	16	80	96	58	580	13.9451	6.7539	12.0172 (86%)	4.73	2837
